# PBX/Knotted 1 homeobox-2 (PKNOX2) is a novel regulator of myocardial fibrosis

**DOI:** 10.1038/s41392-024-01804-5

**Published:** 2024-04-22

**Authors:** Liang Chen, Haotong Li, Xiaorui Liu, Ningning Zhang, Kui Wang, Anteng Shi, Hang Gao, Deniz Akdis, Ardan M. Saguner, Xinjie Xu, Elena Osto, Willem Van de Veen, Guangyu Li, Antoni Bayés-Genís, Firat Duru, Jiangping Song, Xiangjie Li, Shengshou Hu

**Affiliations:** 1 State Key Laboratory of Cardiovascular Disease, Fuwai Hospital, National Center for Cardiovascular Diseases, Chinese Academy of Medical Sciences and Peking Union Medical College, Beijing, P. R. China; 2 School of Statistics and Data Science, Nankai University, Tianjin, China; 3 Department of Cardiology, University Heart Center, University Hospital Zurich and University of Zurich, Zurich, Switzerland; 4 Institute for Clinical Chemistry, University Hospital Zurich and University of Zürich, Zurich, Switzerland; 5 Swiss Institute of Allergy and Asthma Research (SIAF), University of Zurich, Davos, Switzerland; 6 Heart Institute, Hospital Universitari Germans Trias i Pujol, Badalona, CIBERCV Spain

**Keywords:** Translational research, Cardiovascular diseases

## Abstract

Much effort has been made to uncover the cellular heterogeneities of human hearts by single-nucleus RNA sequencing. However, the cardiac transcriptional regulation networks have not been systematically described because of the limitations in detecting transcription factors. In this study, we optimized a pipeline for isolating nuclei and conducting single-nucleus RNA sequencing targeted to detect a higher number of cell signal genes and an optimal number of transcription factors. With this unbiased protocol, we characterized the cellular composition of healthy human hearts and investigated the transcriptional regulation networks involved in determining the cellular identities and functions of the main cardiac cell subtypes. Particularly in fibroblasts, a novel regulator, *PKNOX2*, was identified as being associated with physiological fibroblast activation in healthy hearts. To validate the roles of these transcription factors in maintaining homeostasis, we used single-nucleus RNA-sequencing analysis of transplanted failing hearts focusing on fibroblast remodelling. The trajectory analysis suggested that *PKNOX2* was abnormally decreased from fibroblast activation to pathological myofibroblast formation. Both gain- and loss-of-function in vitro experiments demonstrated the inhibitory role of *PKNOX2* in pathological fibrosis remodelling. Moreover, fibroblast-specific overexpression and knockout of *PKNOX2* in a heart failure mouse model induced by transverse aortic constriction surgery significantly improved and aggravated myocardial fibrosis, respectively. In summary, this study established a high-quality pipeline for single-nucleus RNA-sequencing analysis of heart muscle. With this optimized protocol, we described the transcriptional regulation networks of the main cardiac cell subtypes and identified PKNOX2 as a novel regulator in suppressing fibrosis and a potential therapeutic target for future translational studies.

## Introduction

Heart diseases significantly contribute to global public health burdens, standing as major causes of morbidity and mortality worldwide.^1,2^ Achieving advanced therapeutic strategies necessitates a profound understanding of human heart components and molecular regulatory mechanisms. The heart, a complex organ with four morphologically and functionally distinct chambers, requires exploration of the unique gene regulatory networks within each cardiac cell type. This exploration is crucial for elucidating the pathogenesis of cardiovascular diseases and serves as a fundamental step towards alleviating the burden of cardiovascular ailments.

An accurate characterization of the cellular composition of the human heart is a fundamental piece of knowledge that is essential for understanding the changes that take place during heart development, ageing, and many other pathophysiological changes, as well as for developing tissue engineering and organoid approaches. The major functional component of the heart is the myocardium, defects in which can cause pathological remodelling, such as heart failure, affecting millions of patients around the world.^3^ Multiple attempts have been made to define cell proportions in the heart, but the results remain controversial. The myocardium has a complex cellular architecture consisting of cardiomyocytes (CMs) and nonmyocytes. CMs are the major functional cell type for pumping blood, but they account for only ~30% of the cells in the myocardium.^4^ Nonmyocytes compose the microenvironment to maintain the function of the myocardium and participate in pathological remodelling processes, such as myocardial fibrosis. Although these cells are not functional cells for cardiac contraction, they play a crucial role in maintaining the physiological balance of the heart and are becoming increasingly a focus of research in the field of cardiovascular disease. There is less consensus on the proportion of cardiac nonmyocytes from previous studies using traditional known marker gene labelling techniques limited by the accuracy and depth of detection. Fibroblasts have long been believed to be the largest cell population of nonmyocytes. However, a recent study utilizing multilabelled flow cell analysis showed that ECs account for most nonmyocytes (64%).^4^ Further research is required to clarify the exact composition of cardiac cells. For such a complex and heterogeneous organ like the heart, its various functions are jointly regulated by CMs and nonmyocytes. When this intricate coordination becomes disordered, it can lead to abnormal states in the heart and result in disease. Therefore, elucidating the cellular heterogeneities and their regulatory mechanisms in normal and diseased hearts is of great importance for treating heart-related diseases.

Single-cell transcriptomics has revolutionized our understanding of cell composition and associated gene expression of the human heart. Emerging single-cell RNA sequencing (scRNA-seq) technology has been used to comprehensively characterize cardiac development, homeostasis, and diseases.^5–7^ Building a human cell atlas requires expression profiling of human tissues from different samples. However, three major obstacles prevent the broader application of this technology in studying heart tissues: the difficulty in isolating CMs, artefacts in stress-induced gene activation, the large size of CMs being unsuitable for many high-throughput scRNA-seq platforms, and most importantly, multinucleation of some CMs. To overcome these limitations, large-scale single-nucleus RNA-sequencing (snRNA-seq) has been applied to profile gene signatures of cell heterogeneities in heart muscle.^5,8–13^ Current nuclear isolation and sequencing protocols vary across institutions, and processes for systemic evaluations of sequencing quality, bias, and resolution in myocardial tissue, as well as comparisons with scRNA-seq, remain to be defined. More importantly, due to the difficulties in processing myocardial tissue for nuclear isolation, the gene numbers detected by existing nuclear sequencing have not been as ideal as those detected by intact cell profiling. As a result, the transcriptional regulation networks of the cardiac cell subtypes governing cell identities and homeostasis have seldom been well described in previous studies because of the limited detection of transcription factors (TFs) at low abundance. TFs, as one of the groups of proteins that read and interpret the genetic “blueprint” in the DNA and bind to the DNA and help initiate a program of increased or decreased gene transcription. As such, they are vital for many important cellular processes in the heart like cardiac fibrosis, cardiac rhythm disturbance, CMs apoptosis, and many others. The detection of TFs from the high-depth sn RNA-seq can provide deeper insight into the normal cardiac pathological and physiological status.

Herein, we developed a robust and unbiased tissue processing and single-nucleus transcriptomic profiling pipeline that improved the detection of more TFs and genes involved in signalling pathways. With this optimized protocol, we characterized the cellular composition of healthy hearts and identified the transcriptional regulation networks governing the identities of cell subtypes. A substantial number of TFs were identified to be responsible for cellular identification and homeostasis maintenance of each cell type, among which a novel regulator, PKNOX2, was demonstrated to have fibrosis inhibition potency during fibroblast activation. The in vivo study confirmed the therapeutic effect of enhancing PKNOX2 on alleviating myocardial fibrosis and cardiac dysfunction.

## Results

### Optimized pipeline for cell profiling and TF detection

In the present study, we first established a tissue processing pipeline for nuclear isolation followed by snRNA-seq. The nucleus suspension obtained with our protocol presented an intact morphology with few doublets/triplets (0.9–2.0%) (Supplementary Fig. 1a-c) and a high level of purification (92.8%) (Supplementary Fig. 1d). The RNA integrity number (RIN) from the purified nuclei was similar to that of the tissue before nuclei isolation (RIN: 7.8 vs. 8.1) and better than FACS sorting nuclei (mean RIN: 7.0 vs. 4.8, *p* < 0.0001), suggesting a minimal effect on RNA quality by the optimized isolation process, indicating the potential of this technique in obtaining high-quality transcriptome results (Supplementary Fig. 1e, f).

With the optimized protocol, we performed snRNA-seq using a 10X genomics chromium platform with different reagents and compared it with traditional enzymatic digestion-based scRNA-seq and results in previously published datasets (Supplementary Fig. 2a). The scRNA-seq used fresh tissue sample and the snRNA-seq used frozen heart tissue. The sequencing reads obtained via snRNA-seq contained large proportions of intronic and intergenic regions (Supplementary Fig. 2b). When we used both mapped exon and intron reads for gene detection, the number of detected genes per cell obtained by the nuclear sequencing 3’ V3 pipeline was substantially improved compared to the other nuclear sequencing pipelines and was comparable or even superior to scRNA-seq (Supplementary Fig. 2c). In addition, nuclear sequencing techniques presented fewer mitochondrial transcripts (Supplementary Fig. 2c). All four pipelines identified all nine major cardiac cell types, except that CMs could not be well detected by scRNA-seq techniques because of cell damage during enzyme digestion (Supplementary Fig. 2d). With the optimized pipeline using the 3’V3 reagent, we compared our results with four previously published datasets,^5,9,11,14^ and identified a significantly higher number of detected genes (2501 vs. 815/1102/1386/1603), more than double the number of UMI counts (8801 vs. 1456/2190/3029/2538), and significantly higher numbers of detected TFs (541 vs. 60/64/134/162) (Supplementary Fig. 2e).

To compare the transcriptomes obtained via nuclear and whole-cell profiling, the scRNA-seq and snRNA-seq datasets obtained with the same 3’V2 reagent were used for analysis, with the focus mainly on shared nonmyocyte populations (Supplementary Fig. 3a). Each cell type shared similar marker genes between both datasets (Supplementary Fig. 3b) and exhibited favourable consistency in cell identification (Supplementary Fig. 3c). In particular, we identified a novel pericyte subtype at the border between smooth muscle cells (SMCs) and pericytes through snRNA-seq, which was not observed in the scRNA-seq dataset (Supplementary Fig. 3d). Between these two datasets, 1635 genes (28.5%) were differentially detected, including mitochondrial and ribosomal genes enriched in scRNA-seq and long noncoding RNAs (lncRNAs) preferentially detected in snRNA-seq (Supplementary Fig. 3e, f). Stress-stimulated genes were extensively increased in the intact-cell sequencing dataset due to enzymatic digestion at 37 °C during cell isolation (Supplementary Fig. 3g).

We then compared the preferentially detected genes of nuclei and cellular sequencing in fibroblasts and endothelial cells (ECs) (Supplementary Fig. 3h, i). In both fibroblasts and ECs, the genes found to be enriched in the scRNA-seq analysis were mainly involved in the mitochondrial oxidative phosphorylation (OXPHOS) pathway, in addition to stress-induced cell damage and inflammation pathways, whereas the protein phosphorylation cascade and cellular signal transduction pathways were found to be enriched in the snRNA-seq analysis. Intact-cell profiling exhibited high sensitivity in detecting immune-related cellular surface antigens and receptors such as *HLA* genes and *CXCL/CCL* genes, while nuclear-based profiling performed better in detecting TFs of fibroblasts and vasculature with high abundances of collagen genes, such as *FOXO1*, *MEOX2*, *NF1*, and *RORA*, in ECs. These results suggest the advantage of studying cellular signalling pathways and transcriptional regulation by means of snRNA-seq. However, we need to clarify that these differences between scRNA-seqand snRNA-seq could come from both fresh and frozen tissue differences as well as single-cell and single-nucleus technical comparisons. In brief, our optimized snRNA-seq techniques applied to adult human hearts achieved gene detection results superior to those obtained with scRNA-seq, with favourable robustness and repeatability.

### Characterizing the cellular composition in healthy hearts

**Fig. 1:**
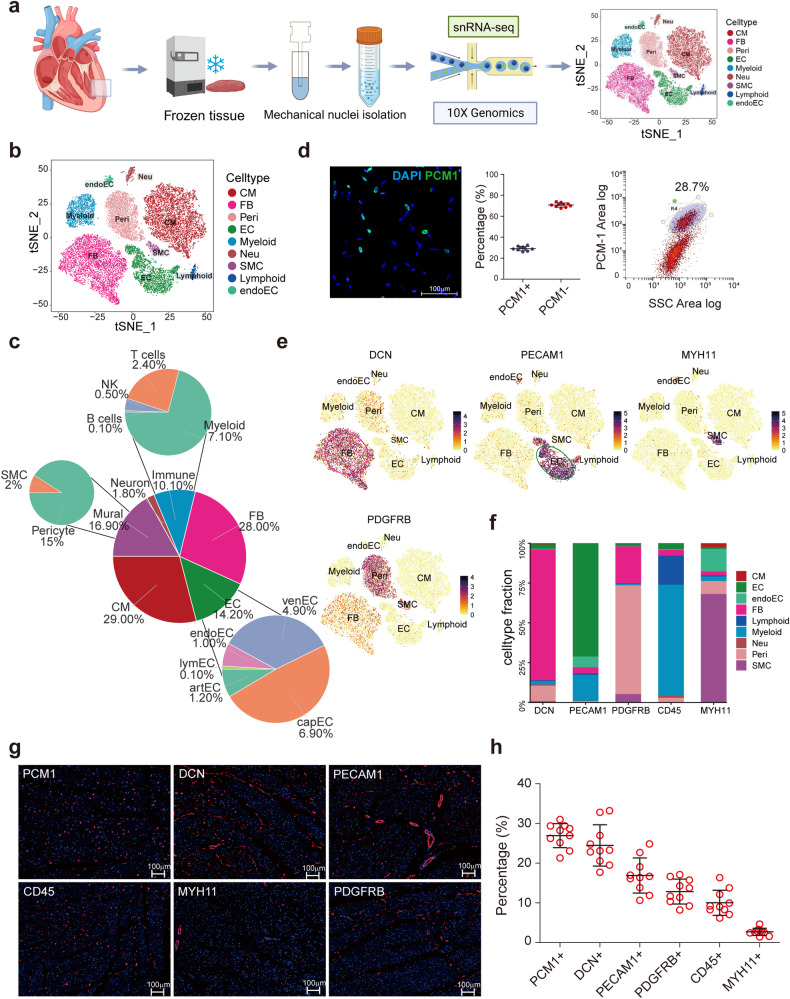
Unbiased characterization of human cardiac cellular composition. **a** snRNA-seq workflow using the 10X Genomic platform with five human donor hearts (*n* = 5, donor #2–#6 shown in Table S1, male, age 29–50). **b**TSNE plot showing the distribution of cell types for snRNA-seq and scRNA-seq. **c** Composition of cell culsters and subclusters of immune cell and EC.**d** The proportion of cardiomyocyte nuclei in human left ventricular myocardium was verified by immunostaining and flow cytometry, and both PCM1 and DAPI positive were cardiomyocyte nuclei (*n* = 5, two independent runs from each donor, and the same 5 donors enrolled shown in Table S1). The left panel: immunostaining representative figure of cardiac nuclei, blue, DAPI; green, PCM1. The middle panel, quantitative analysis of the proportions of PCM1+ (cardiomyocyte) and PCM1- (non-cardiomyocyte) nuclei. The right panel, flow cytometry representative figure shows PCM1+ nuclei percentage (**e**) Cell-type-specific marker gene assessment, DCN for FB, PECAM1 for EC, MYH11 for SMC and PDGFRβ for pericyte. **f** The expression ratios of five major non-cardiomyocyte marker genes in different cells. **g** Fluorescence immunohistochemistry staining to validate the proportions of major heart cell populations: PCM1+ cardiomyocytes; DCN+ FBs; PECAM+ECs; PDGFRB+ pericytes; CD45+ immune cells; and MYH11+ SMCs. **h** Quantitative analysis of the proportions of cells expressing marker genes by automatic imaging segmentation and quantitation. Ten sections from the ventricles of five hearts (two sections from each heart, 5 donors enrolled shown in Table S1) were measured. CM, cardiomyocyte; FB, fibroblast; Peri, pericyte; EC, endothelial cell; Neu, neurone; SMC, smooth muscle cell; endoEC, endocardial endothelial cell; DAPI, 4’,6-diamidino-2-phenylindole

With the optimized protocol, we performed snRNA-seq for five healthy donor hearts (donors #2–#6, sample information in Supplementary Table S1), followed by experimental validation through flow cytometry and immunostaining, to quantify the cell composition in the hearts (Fig. 1a). Nine major cardiac cell types and their proportions were defined by distinct gene signatures (Fig. 1b, c, Supplementary Table S2). The percentage of cardiomyocyte nuclei in the ventricular myocardium was approximately 29%, consistent with the findings of previous reports.^4,15^ This result was verified by flow cytometry to evaluate nuclei positive for the cardiomyocyte nuclear marker *PCM-1* (Fig. 1d). As 20–30% of human CMs are binucleated,^15^ the exact proportion of CM cells cannot be well determined by present protocol. Our data showed that fibroblasts accounted for the largest portion of nonmyocytes, with 28.0% of total nuclei. ECs (including endoECs) accounted for 14.2% of the total nuclei. We observed an unexpectedly high proportion (15%) of pericytes, which was much higher than the previously reported 5% in the murine heart by in situ analysis.^4^ The ratio of pericytes to ECs was ~1:1, much higher than previous observations of 1:2–1:3 from bovine hearts^16^ and 1:4–1:5 from murine hearts.^4^ Immune cells accounted for 10.1% of all nuclei. In addition, we utilized our previously published CD45^+^ immune cell-enriched scRNA-seq to quantify the composition of immune cells,^7^ and the results revealed that myeloid cells were the major component (70.3%) of immune cells, T lymphocytes accounted for 23.7% of immune cells, and B cells accounted for 1.0% of immune cells.

Moreover, we screened the cell-specific marker genes to validate the cell proportions by in situ histology (Fig. 1e). The immune cells and ECs were well represented by exclusive pan-markers, such as *CD45*, representing immune cells, and *PECAM1*, representing ECs. Although exclusive marker genes were not well recognized for fibroblasts, pericytes, and SMCs, we identified markers with relatively high specificities (>75%) for the identification of these cell types, including *DCN* (for fibroblasts), *PGDFRB* (for pericytes), and *MYH11* (for SMCs) (Fig. 1f). Using these markers, we performed in situ immunofluorescence and quantified the proportions of cells expressing each marker gene (Fig. 1g, Supplementary Fig. 4). The quantification of positive cells indicated consistency with the cellular proportions observed via snRNA-seq (Fig. 1h).

### Vascular cell populations and transcriptional regulation in healthy hearts

**Fig. 2:**
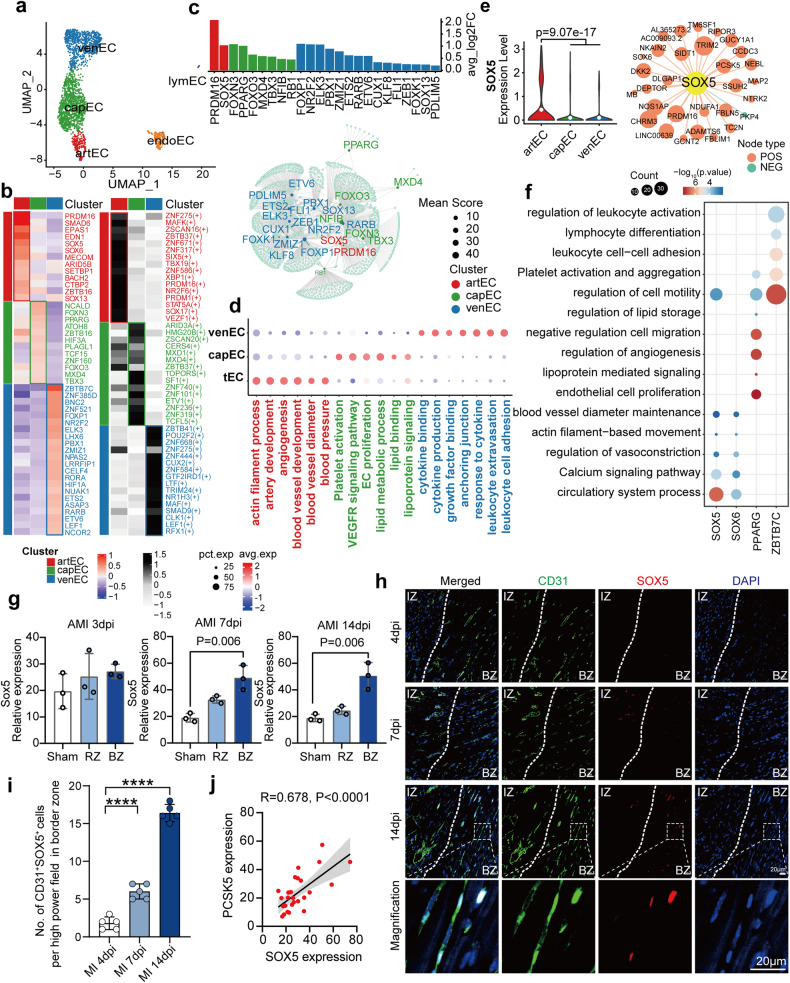
Endothelial cell subpopulations and gene regulation analysis. **a** UMAP plot showing the five subclusters of ECs, including vascular ECs, endoECs, lymECs. Vascular ECs consist of artECs, capECs, and venECs. **b** Top TF and regulons expression in each vascular EC. Left panel: heatmap shows the relative expression of the top TF genes in each vascular EC subtype; right panel: heatmap shows the normalized activity of the top TF regulons in each vascular EC subtype predicted by pySCENIC. **c** The overlapping TFs in terms of the expression and predicted activity analysis in (**b**). Upper panel: Bar graph showing the top differentially expressed TFs in each vascular EC subtype; lower panel: Network representation of selected differentially expressed TFs among vascular EC subtypes, as analyzed by pySCENIC. **d** Enriched pathways associated with vascular EC subtype-specific TFs. These pathways indicate that subtype-specific TFs govern genes responsible for vascular functions. **e** The expression levels and target gene networks of representative novel TFs in artEC subtype (SOX5). **f** Enriched pathways of the targeted genes of these representative TFs (SOX5, SOX6, PPARG, ZBTB7C). **g** Validation of *SOX5* expression in the bulk transcriptome dataset (GSE110209) of AMI murine model which includes remote zone and border zone at 3, 7, 14 dpi. **h** Immunostaining illustrates SOX5 (red) expression increased in endothelial cells (CD31, green) of border zone after acute myocardial infarction (AMI) in wild-type mice at 4, 7, 14 dpi. Scale bars, 20 μm. **i** Number of both SOX5 and CD31 positive cells in one high power field of border zone at 4, 7, 14 dpi. **j** Pearson correlation of *SOX5* and the arteriole marker gene *PCSK5* in the same dataset. EC, endothelial cell; endoEC, endocardial endothelial cell; lymEC, lymphatic endothelial cell; artEC, arteriole endothelial cell; capEC, capillary endothelial cell; venEC, venule endothelial cell; TF, transcription factor; AMI, acute myocardial infarction; dpi, day post-infarction; RZ, remote zone; BZ, border zone. Data are presented as mean ± SD

Our previous study demonstrated functional zonation of vascular cells, including ECs and mural cells.^10^ In the present study, we focused on illuminating the transcriptional regulation networks responsible for determining the fate of vascular cells from artery to vein zonation. ECs were classified into five subtypes: three types of vascular ECs, endocardial cells (endoECs), and lymphatic ECs (lymECs) (Fig. 2a, Supplementary Table S3). Vascular ECs were clustered into three major subtypes along the artery-capillary-vein axis, with distinct gene signatures and functional pathways, as we previously reported^10^ (Supplementary Fig. 5a–c). Both differentially expressed TFs and transcriptional regulon activity analysis by single-cell regulatory network inference and clustering (SCENIC) analysis demonstrated distinct regulatory networks among the three vascular ECs (Fig. 2b). The overlap of these two TFs sets was plotted in Fig. 2c. The most remarkable regulon of arteriole ECs (artECs) was *PRDM16*, which was recently demonstrated to coordinate angiogenesis^17^ and maintain artery flow function^18^ (Supplementary Fig. 5d). Furthermore, most detected artEC-enriched TFs have been previously demonstrated to be involved in angiogenesis and blood pressure regulation, including *SMAD6*,^19^ *EPAS1*,^20^ *EDN1*,^21^ *MECOM*,^22^ and *MAML3*^23^ (Fig. 2b, d). In the present study, capillary ECs (capECs) were regulated by *NFIB*, the upstream regulator of vWF that was highly expressed in capECs (Supplementary Fig. 5d, e). The co-expressed genes of capEC-enriched TFs were associated with EC proliferation and lipid transport functions (Fig. 2d). Postcapillary venule ECs (venECs), which contribute to leucocyte trafficking and permeability in the disease state, expressed genes associated with cytokine binding and regulation of cell migration that were regulated by distinct TFs, such as *NR2F2* and *FOXP1*. FOXP1 has been demonstrated to be a gatekeeper of EC inflammation inversely correlated with potential target genes associated with immune cell extravasation^24^ (Fig. 2d, Supplementary Fig. 5d). The pathways enriched in co-expressed genes of TFs indicated the individual functions of the three types of ECs, suggesting the essential roles of TFs in vascular homeostasis (Fig. 2d).

In addition, our present study also identified some novel TF candidates, such as *SOX5* and *SOX6* in artECs, *PPARG* in capECs, and *ZBTB7C* in venECs (Fig. 2e, Supplementary Fig. 5f, Supplementary Table S4). Their potential targeted genes were strongly associated with the functions of their corresponding EC subtypes (Fig. 2f). We further examined the expression alterations of these TFs in disease models. In the published RNA-seq dataset (GSE132146) of human explanted hearts with chronic myocardial infarction (CMI),^25^ *ZBTB7C* expression was significantly increased and correlated with the expression of lymphocyte adhesion molecules of ECs (*ICAM1* and *CDH5*), suggesting a potential function in the regulation of vascular inflammation for *ZBTB7C* (Supplementary Fig. 5g). In the transcriptome analysis (GSE110209) of an acute myocardial infarction (AMI) murine model,^26^ *Sox5* expression was significantly increased in the border zone after 14 days post-AMI (Fig. 2g) and was validated by immunofluorescence (Fig. 2h, i, Supplementary Table S5, 6), which was positively correlated with *Pcsk5*, the marker gene of arteriole vessels (Fig. 2j). *Sox6* and *Pparg* expression was also studied in the AMI murine transcriptome data (Supplementary Fig. 5h). These results suggested a potential function of *SOX5* in arteriogenesis during AMI.

**Fig. 3:**
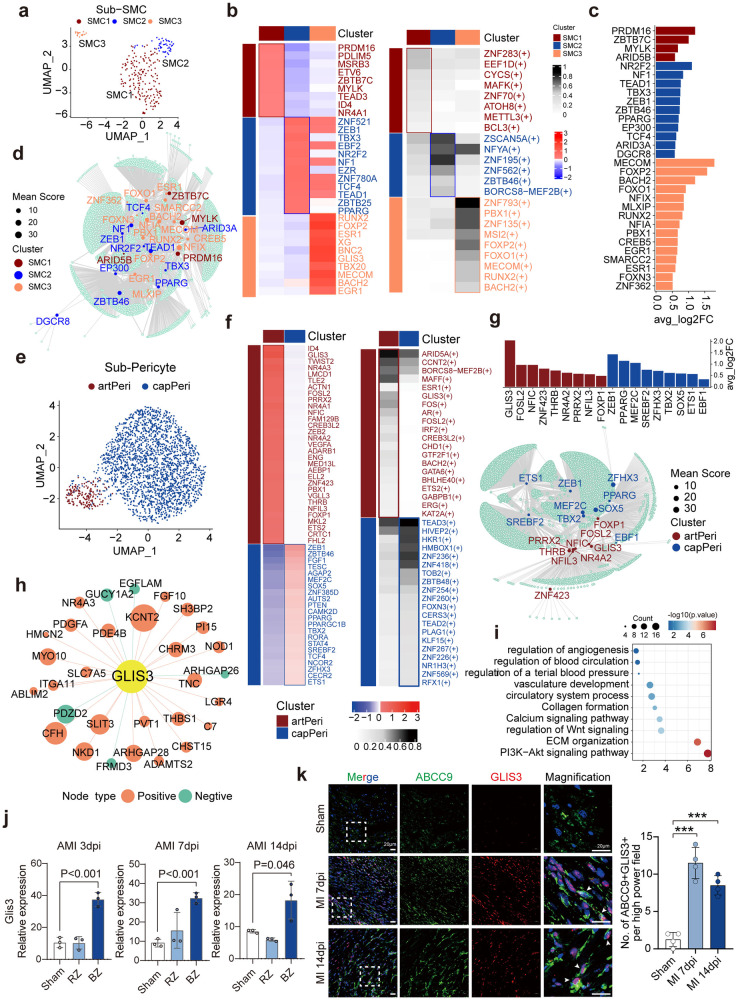
Smooth muscle cells and pericyte subpopulations and gene regulation analysis. **a** UMAP plot showing the three subclusters of SMC. **b** Top TF and regulons expression in each subcluster of SMC. Left panel: heatmap shows the relative expression of the top TF genes in each SMC subtype; right panel: heatmap shows the normalized activity of the top TF regulons in each SMC subtype predicted by pySCENIC. **c**–**d** The overlap TFs (**c**) and their regulatory networks (**d**) in each SMC subtype. (**e**) UMAP plot showing the two subclusters of pericytes. (**f**) Top TF and regulons expression in each subcluster of pericyte. Left panel: heatmap shows the relative expression of the top TF genes in each pericyte subtype; right panel: heatmap shows the normalized activity of the top TF regulons in each pericyte subtype predicted by pySCENIC. **g** The overlap TFs and their regulatory networks in each pericyte subtype. **h**, **i** Targeted genes of the TF *GLIS3* (**h**) and the enriched pathways of those target genes (**i**). **j** Validation of *GLIS3* expression in the transcriptome dataset of acute myocardial infarction murine model (GSE110209) which includes remote zone and border zone bulk RNA-seq data at 3, 7,14 dpi. **k** Immunostaining illustrates GLIS3 (red) expression increased in pericytes (ABCC9, green) after AMI in wild type mice at 7 dpi and 14 dpi (left panel); number of both SOX5 and CD31 positive cells in one high power field of border zone at 4, 7, 14 dpi (right panel). Scale bars, 20 μm. SMC, smooth muscle cell; avg_log2FC, average log2 fold change; TF, transcription factor; artPeri, arteriole pericyte; capPeri, capillary pericyte; AMI, acute myocardial infarction; dpi, day post-infarction; RZ, remote zone; BZ, border zone. Data are presented as mean ± SD

The SMCs were mainly divided into three subtypes: arterial SMCs (artSMCs), defined by muscle structure development genes; venule SMCs (venSMCs), defined by genes involved in cell migration and motility; and undefined SMCs, enriched in genes associated with extracellular matrix organization **(**Fig. 3a, Supplementary Fig. 6a-c, Supplementary Table S7). We investigated the differential expression of TFs and the transcriptional regulon activities of TFs by SCENIC in SMC subtypes (Fig. 3b). The overlapping TFs were identified (Fig. 3c), and their regulatory networks are presented in Fig. 3d. These subtype-specific SMC TFs indicated synergic functions with ECs along the vasculature. *PRDM16*, a star regulator of artEC, as previously mentioned,^17,18^ was also significantly enriched in artSMC, indicating that *PRDM16* may govern coronary artery/arteriole homeostasis at multiple cell levels. Similarly, venSMC2 also shared some transcriptional regulons with venEC, such as *NR2F2*. The undefined SMC was regulated by *RUNX2*, *FOXP2*, and *BACH2*, some of which were recently reported to be involved in cardiac homeostasis and vascular calcification.^27^ The function and definition of this SMC subtype remain to be elucidated.

Pericytes were mainly divided into two subtypes: arteriole pericytes (artPeris), enriched in ECM and muscle structure genes, and capillary pericytes (capPeris), defined by genes involved in blood vessel development and lipid metabolism (Fig. 3e, Supplementary Fig. 6d–f, Supplementary Table S8).^10^ We investigated the differential expression of TFs and the transcriptional regulon activities of TFs by SCENIC in pericyte subtypes (Fig. 3f). Their overlapping TFs were identified, and their regulatory networks are presented in Fig. 3g. These TFs suggest interesting functions, such as artPeri-enriched *PRRX2* and *NR4A2* involved in vascular homeostasis^28,29^ and capPeri-enriched *PPARG* and *TBX2* coexpressed with capillary ECs.^30^ In particular, *GLIS3* showed significantly increased expression and high regulon activities in artPeris. Moreover, its targeted genes were enriched in collagen formation and angiogenesis (Fig. 3h, i). We established an AMI murine model and confirmed that *GLIS3* significantly increased in the border zone from the early-stage post-AMI, consistent with the angiogenesis process after myocardial injury (Fig. 3j, k, Supplementary Fig. 6g). As pericytes are understudied, the regulatory functions of these novel TFs remain to be investigated to illuminate the pivotal role of pericytes in cardiovascular homeostasis.

### Fibroblast populations and transcriptional regulation in healthy heart

**Fig. 4:**
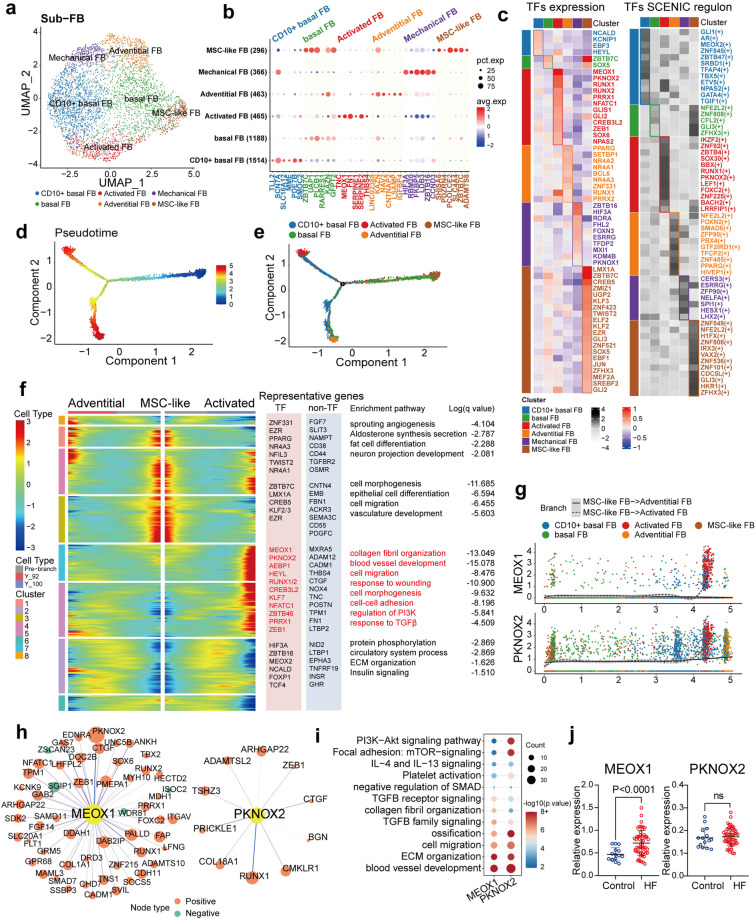
Defining fibroblasts populations and cell transformation within the human heart. **a** UMAP plot showing the six subclusters of fibroblasts. **b** Dot plot showing the representative marker genes of each subtype of fibroblasts. **c** Left panel: heatmap shows the relative expression of the top transcription factor (TF) genes in each fibroblast subtype; right panel: heatmap shows the normalized activity of the top TF regulons in each fibroblast subtype predicted by pySCENIC. **d**, **e** Pseudotime analysis of fibroblasts (**d**) and analysis coloured by fibroblast subtypes (**e**). **f** Heatmap showing the gene expression pattern along two different trajectories (from MSC-like FB to adventitial FBs and from MSC-like FB to activated FBs), accompanied by representative TF and non-TF genes and enriched pathways in different modules. **g** Gene expression patterns of *MEOX1* and *PKNOX2* along trajectories associated with fibrogenesis (high expression in activated FBs). **h** Targeted genes of the TFs *MEOX1* and *PKNOX2* predicted by SCENIC. The degree of correlations of TF and targeted genes are represented by the size of the circle, red means positive correlation and green means negative correlation. **i** Enriched pathways of genes related to representative TFs in the activated FB subpopulation. **j** Expression levels of *MEOX1* and *PKNOX2* in explanted human hearts with heart failure (HF, *n* = 52) and controls (*n* = 15) (data from GSE145154). FB, fibroblast; MSC, mesenchymal stem cell; TF, transcription factor; HF, heart failure; ns: not significant. Data are presented as mean ± SD

Subclustering analysis of fibroblasts identified six subtypes within the human heart (Fig. 4a, b, Supplementary Fig. 7a, b, Supplementary Table S9). Two major clusters were defined as basal subtypes: one expressing the cell surface antigen *CD10* (encoding neprilysin) and the other without specific marker genes (Supplementary Fig. 7c). Activated fibroblasts (activated FBs) were identified by *POSTN* and fibrotic collagens *COL1A2* and *COL3A1* (Fig. 4b, Supplementary Fig. 7c), whereas myofibroblasts (myoFBs) were excluded on the basis of the lack of expression of *ACTA2*. Activated FBs exhibited high expression of the fibrogenic TFs *MEOX1* and *AEBP1*, as we previously reported (Fig. 4c, Supplementary Fig. 7d).^7^ Adventitial fibroblasts (adventitial FBs) expressed genes involved in the autonomic nervous system (i.e., *KCND2* and *NAV2*) at high levels and secreted many ligands for sprouting angiogenesis, such as *ANGPT1*, *VEGFC*, and *FGF7* (Fig. 4b, Supplementary Fig. 7a–c). We further examined the cellular expression and correlations of genes involved in these two distinct pathways and confirmed that this cell subtype simultaneously exhibits these two functions (Supplementary Fig. 7e). Adventitial fibroblasts highly expressed the TFs *NR4As* (e.g., *NR4A1/2/3*) and *PPARG* (Fig. 4c, Supplementary Fig. 7d), suggesting potential roles in not only vascular angiogenesis but also perivascular adipogenesis for these cells, which was consistent with the finding that adipose tissue is enriched in perivascular regions in the human heart.^31^ In addition, adventitial FBs showed the most significant correlation with artPeris among all fibroblast subtypes, suggesting synergistic gene signatures and functions as perivascular cells (Supplementary Fig. 7f). A small subpopulation (6.9%) of fibroblasts was identified as mesenchymal stromal cell-like FBs (MSC-like FBs) due to their pluripotency, as shown in the enriched pathways (Supplementary Fig. 7a, b). Myocardial MSC-like FBs were possible to distinguish by the unique expression of *SHISA6* and the long noncoding RNA *LINC01133* (Fig. 4b, Supplementary Fig. 7c), both of which are understudied in the context of heart physiology. However, they have both been demonstrated to inhibit the canonical Wnt/β-catenin pathway and promote cell proliferation and self-renewal in cancer^32^ and the reproductive system.^33^ Many TFs have been identified in MSC-like FBs, including *LMX1A* and the repressor *KLF3* (Fig. 4c, Supplementary Fig. 7d). All these TFs perform functions in cell fate determination^34–36^ but have not been studied in cardiac MSC-like FBs. We also identified a subtype of fibroblasts, namely, ‘mechanical fibroblasts’, based on the high expression of cardiomyocyte-derived heart muscle contraction genes (Fig. 4a, b, Supplementary Fig. 7a, b). This subtype has been reported in published single-cell and nucleus sequencing studies of heart tissue,^5^ but ambient RNA contamination remains a critical issue to be evaluated. We further identified the top activities of the TF regulatory networks (regulons), some of which were consistent with differentially expressed TFs, in each subtype by SCENIC (Fig. 4c).

We then aligned fibroblasts in pseudotime to model cell remodelling. The predicted trajectory illuminated the directions from MSC-like FBs to two differentiated phenotypes, adventitial FBs and activated FBs (Fig. 4d, e). The cell lineage of adventitial FBs showed increased expression of angiogenesis-related secreted ligands (*FGF7*, *VEGFC*, and *SLIT3*) and novel receptors (*OSMR* and *TGFBR2)*, as well as TFs associated with vascular development (*NR4A1/3*) and the mesodermal cell regulator *TWIST2* (Fig. 4f, Supplementary Table S10). Adipogenesis was also enriched in adventitial FBs according to the enriched pathways and high expression of *PPARG* (Fig. 4f). Analysis of differentially expressed genes during activated FB development showed that in addition to a set of fibroblast activation markers (*POSTN*, *TPM1*, *FN1*, and *LTBP2*) and common fibrogenic genes (*THBS4*, *CTGF*, and *NOX4*), the expression levels of genes with antifibrotic properties, such as *MXRA5* and *ADAM12*,^37^ were also increased, which might restrict the fibrogenic cascade in the normal heart (Fig. 4f). We further screened TFs and identified the most significantly activated TF as *MEOX1*^38^ in activated FBs, followed by the previously unreported *PKNOX2* (Fig. 4g). Interestingly, these TFs showed regulatory relationships with each other according to transcriptional regulon analysis (Fig. 4h). *MEOX1* was positively correlated with *PKNOX2*, whereas *PKNOX2* potentially regulated *RUNX1* and the fibrogenic cytokine *CTGF*. Furthermore, the pathways in which genes correlated with these TFs were enriched were pathways such as collagen organization and TGFB signalling (Fig. 4i). We further validated these TFs in an independent bulk transcriptome dataset (GSE145154) previously published by our group that contains data for 15 normal donors and 52 transplanted failing hearts.^7^ The results indicated that *MEOX1* was highly expressed in the context of heart failure, whereas *PKNOX2* was not increased in bulk RNA profiling, suggesting a different pathogenic role of *PKNOX2* (Fig. 4j).

### Transcriptional dysregulation of *PKNOX2* during fibrosis in failing hearts

**Fig. 5:**
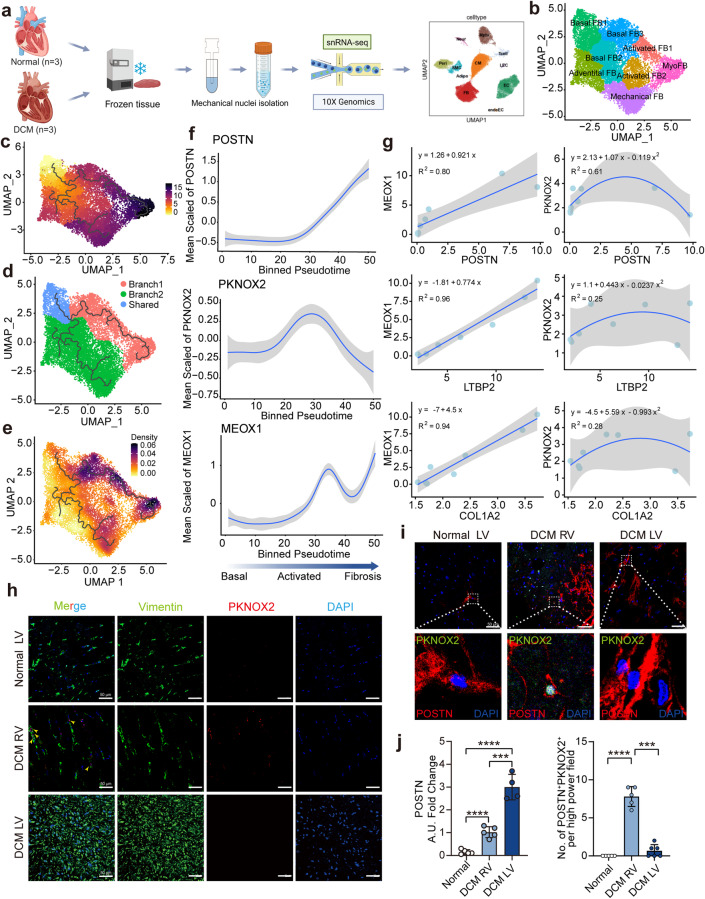
Alterative expression of PKNOX2 during fibroblasts remodelling trajectory within the failing human heart. **a** snRNA-seq workflow of healthy (*n* = 3) and transplanted human hearts (*n* = 3) with dilated cardiomyopathy (DCM) using the 10X Genomic platform. **b** UMAP plot showing the 8 subclusters of fibroblasts. **c** Pseudo-time analysis of fibroblasts by monocole 3. **d** The trajectories of fibroblasts were mainly divided into two branches. **e** The average expression levels of gene involved in collagen fibril organization indicated branch 1 as a representative fibrosis formation path. **f** The gene expression of POSTN, PKNOX2, and MEOX1 along the trajectory branch 1 of fibroblasts from basal state to fibroblast activation then to fibrosis. **g** MEOX1 and PKNOX2 expression correlation with selected fibrosis marker genes, MEOX1 was linearly correlated with fibrotic genes, while PKNOX2 was nonlinearly correlated with fibrotic genes. **h** Immunofluorescence of PKNOX2 expression in left ventricle from healthy hearts, right ventricular (RV) and left ventricular (LV) of DCM hearts. Yellow arrows indicate PKNOX2 expressed in fibroblast which identified by mesenchymal marker Vimentin. **i** Co-localization of PKNOX2 and POSTN in left ventricle from healthy *n* = 5, RV of DCM (*n* = 5), and LV of DCM hearts (*n* = 4). Yellow arrows indicate PKNOX2 positive. The below figures are ×10 magnification of the region of interest in the above figures. **j** Average fluorescence intensity of POSTN in these three groups (left panel). Number of both PKNOX2 and POSTN positive cells in one high power field among three groups (right panel). A.U.: Arbitrary Unit. DCM, dilated cardiomyopathy; RV, right ventricular; LV, left ventricular; DAPI, 4’,6-diamidino-2-phenylindole. Data are represented as mean ± SD. ****P < 0.0001

Considering that bulk gene profiling might conceal the changes in *PKNOX2* in individual cells, we explored fibroblast remodelling in transplanted failing hearts with dilated cardiomyopathy (DCM, *n* = 3) in comparison to healthy controls (*n* = 3) with optimized snRNA-seq (Fig. 5a). Unsupervised clustering identified 8 fibroblast subclusters with distinct gene signatures and enriched functions (Fig. 5b, Supplementary Fig. 8a, b, Supplementary Table S11). Fibroblasts from heart failure patients not only shared basal subclusters with healthy controls but also had a substantial number of new subtypes (Supplementary Fig. 8c). In particular, an additional distinct subtype was identified as myoFBs with pathways enriched in supramolecular fibre organization in the failing hearts (Supplementary Fig. 8a, b). MyoFBs exhibited the highest expression of fibrosis marker genes, such as *COL1A1*, *COL1A2*, *POSTN*, *LTBP2*, and *THBS4*. When we transferred the cell identifications of this disease dataset using the six subtypes of healthy hearts as the reference, the results showed a favourable consistency with unsupervised classification. Although MSC-like FBs were not defined by unsupervised classification, the transfer-label analysis identified them as a small subcluster within the basal FB1 (Supplementary Fig. 8d). The incremental subtypes from failing hearts were all defined as activated FBs, including myoFBs (Supplementary Fig. 8d). As identified by label-transfer cell identification, activated FBs (including myoFBs) were significantly increased in heart failure patients compared to healthy controls (Supplementary Fig. 8e).

We also performed pseudotime analysis to identify the potential pathological remodelling pathways of fibroblasts (Fig. 5c, Supplementary Fig. 8f), and two main trajectories were observed along MSC-like FBs to diseased FBs, suggesting sophisticated and multidirectional remodelling in heart failure (Fig. 5d). Trajectory 1 showed a gradient increase in the collagen fibril fibrosis score from basal FBs to myoFBs, which might reflect the myocardial fibrosis formation process originating from resident fibroblasts (Fig. 5e). Along this trajectory, the expression of *POSTN* and MEOX1 started to increase at the initiation time of fibroblast activation, whereas *PKNOX2* expression peaked at this time and gradually decreased from activated FBs to myoFBs (Fig. 5f). We also analysis the expression of MSC-like FB marker gene SHISA6 and SLC4A4 expression pattern along the trajectory (Supplementary Fig. 8g). Furthermore, we analysed the expression correlation of TFs and fibrosis-related genes. In contrast to *MEOX1*, which was linearly and positively correlated with fibrosis genes, *PKNOX2* expression had a nonlinear quadratic relationship (Fig. 5g). These results suggested that *PKNOX2* might possess negative regulation, especially at the transition stage from physiological activation to pathological fibrosis.

To validate the rise–fall expression pattern of *PKNOX2* during myocardial remodelling, we examined the protein expression in left ventricle myocardium from donor hearts, left ventricle myocardium from DCM hearts (DCM LV, end-stage fibrosis), and right ventricle myocardium from DCM hearts (DCM RV, intermediate state of fibroblast activation). Left ventricle of DCM showed massive fibrotic infiltration while right ventricle had little cardiac fibrosis,^39^ which was confirmed by the expression of *VIM* and *POSTN* in our experiment (Fig. 5h–j, Supplementary Fig. 8h). Our results showed that PKNOX2 was significantly increased in DCM RV fibroblasts and colocalized with POSTN, but this increase disappeared in DCM LV fibroblasts with severe fibrosis (Fig. 5i, j).

### PKNOX2 restrains pathological myocardial fibrosis

**Fig. 6:**
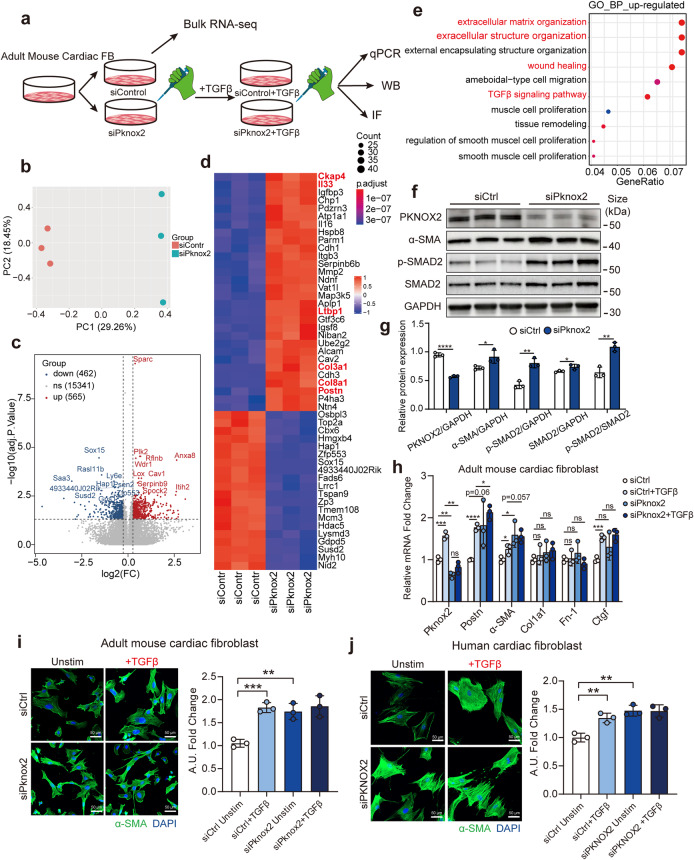
In vitro knockdown of Pknox2 promotes the activation of adult mouse cardiac fibroblasts. **a** Schematic diagram showing the experimental procedure of Pknox2 knockdown and TGFβ stimulation in adult mouse cardiac fibroblast (AMCF). Three independent experiments were performed for each group. Adult mouse ventricular cardiac fibroblasts with SV40 were used. **b** Principal component analysis of expression profile of AMCF with Pknox2-siRNA and scrambled siRNA (*n* = 3 per group). (**c-d**) Volcano plot of differential expressed genes (DEGs) (**c**) and heatmap of top DEGs (**d**) from AMCF with Pknox2-siRNA versus scrambled siRNA. **e** Dot plot showing the functional enrichment of the up-regulated genes in Pknox2-siRNA versus scrambled siRNA. The enrichment significant threshold was set to an adjusted *P*-value < 0.05. **f**, **g** Representative western blots (**f**) and statistical analysis (**g**) of PKNOX2, α-SMA, p-SMAD2, and SMAD2 protein expression in AMCFs isolated from wild-type adult mice with siPknox2 and control siRNA transfected (*n* = 3). **h** Knockdown of Pknox2 in AMCF and stimulated by rmTGF-β, the genes related to fibrosis were detected by RT-qPCR (*n* = 3 per group). **i**, **j** Knockdown of Pknox2 in adult mouse cardiac fibroblast (**i**) and knockdown of PKNOX2 in human cardiac fibroblast (**j**) and then stimulated by rmTGF-β, α-SMA expression was detected by immunofluorescence (green, α-SMA; blue, DAPI). TGFβ, transforming growth factor-beta; rmTGFβ, recombinant mouse transforming growth factor-beta; qPCR, quantitative Polymerase Chain Reaction; IF, immunofluorescence; PC, principal component; Unstim, un-stimulate; ns, not significant; A.U., Arbitrary Unit; DAPI, 4′,6-diamidino-2-phenylindole. Data are represented as mean ± SD. **P < 0.01, ***P < 0.001

To validate the function of PKNOX2 in fibroblast activation and fibrosis formation, we performed loss- and gain-of-function studies in vitro and in vivo. First, *Pknox2* was knocked down by siRNA in immortalized adult mouse cardiac fibroblasts (AMCFs) (Fig. 6a). Through bulk RNA-seq, we identified 565 upregulated and 462 downregulated genes in the Pknox2-siRNA group versus the control group (Fig. 6b, c, Supplementary Table S12). The top differentially expressed genes showed that *Ckap4*, *Il33*, *Ltbp1*, *Col3a1*, *Col8a1*, and *Postn* were upregulated (Fig. 6d), accompanied by enriched pathways such as the TGFβ signalling pathway and extracellular matrix-related terms (Fig. 6e). At the protein level, after knocking down PKNOX2, both the expression and phosphorylation level of SMAD2 were upreglated, along with the increased expression of fibrosis marker a-SMA, suggesting activation of fibrogenic TGF-β-SMAD2 pathways induced PKNOX2 insufficiency. (Fig. 6f, g). In addition, we knocked down *Pknox2* in AMCFs and then stimulated the AMCFs with TGFβ. Surprisingly, the results demonstrated that simple knockdown of *Pknox2* alone could induce fibrosis to the same level of activation as TGFβ stimulation (Fig. 6h). Moreover, TGFβ stimulation did not induce incremental fibrosis activation on the basis of *Pknox2* knockdown (Fig. 6h, i). Similar results were confirmed in human-derived cardiac fibroblasts (Fig. 6j).

**Fig. 7:**
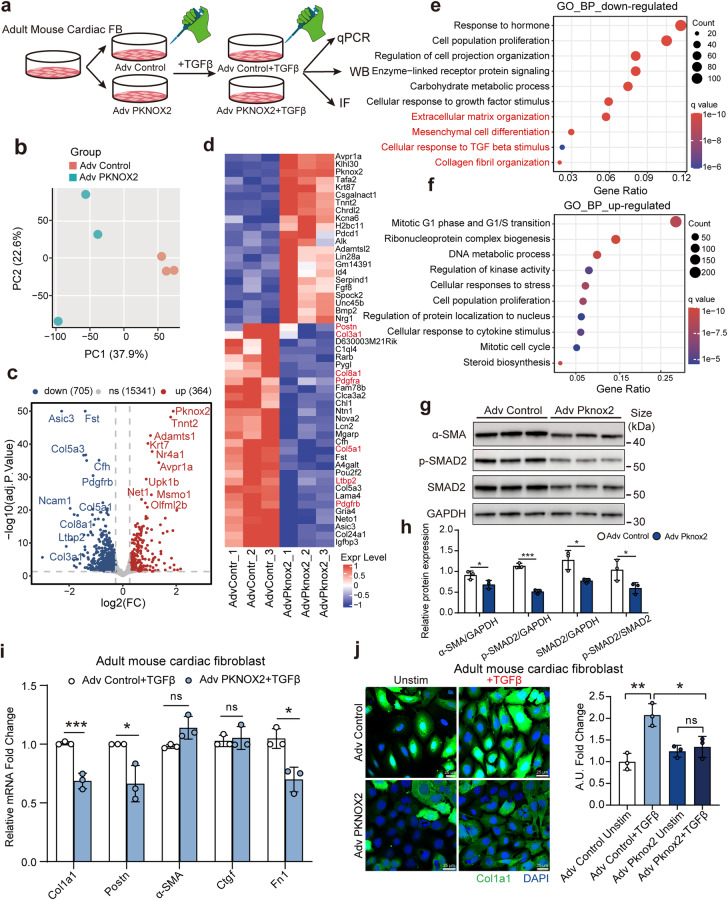
In vitro overexpression of Pknox2 inhibits adult mouse cardiac fibroblasts activation. **a** Schematic diagram showing the experimental procedure of Pknox2 overexpression and TGFβ stimulation in adult mouse cardiac fibroblast (AMCF). (*n* = 3 for each group). **b** Principal component analysis of expression profile of AMCF with Pknox2-siRNA and scrambled siRNA (*n* = 3 per group). **c**, **d** Volcano plot of DEGs (**c**) and heatmap of top DEGs (**d**) from AMCF with Adv-Pknox2 versus Adv-Control. **e**, **f** Dot plot showing the functional enrichment of the down-regulated genes (**e**) and up-regulated genes (**f**) in Adv-Pknox2 versus Adv-Control. The enrichment significant threshold was set to an adjusted *P*-value < 0.05. **g**, **h** Representative western blots (**g**) and statistical analysis (**h**) of, α-SMA, p-SMAD2 and SMAD2 protein expression in AMCFs isolated from wild-type adult mice with Adv-Pknox2 versus Adv-Control (*n* = 3). **i** Overexpression of Pknox2 in AMCF and then stimulated by TGF-β, the genes related to fibrosis were detected by RT-qPCR (*n* = 3 per group). **j** Overexpression of Pknox2 in AMCF then stimulated by TGF-β, Col1a1 expression was detected by immunofluorescence (green, Col1a1; blue, DAPI). TGFβ, transforming growth factor-beta; rmTGFβ, recombinant mouse transforming growth factor-beta; qPCR, quantitative Polymerase Chain Reaction; IF, immunofluorescence; PC, principal component; Unstim, un-stimulate; ns: not significant, A.U.: Arbitrary Unit, DAPI, 4′,6-diamidino-2-phenylindole. Data are represented as mean ± SD. *P < 0.05, **P < 0.01, ***P < 0.001

Furthermore, a gain-of-function experiment was conducted by overexpressing *Pknox2* using adenovirus-delivered plasmid (adv-PKNOX2) infection in AMCFs (Fig. 7a). To examine the inhibitory role of *PKNOX2* in activated FBs, AMCFs were induced by TGFβ as baseline stimulation. Bulk RNA-seq identified 705 downregulated and 364 upregulated genes in the *Pknox2* overexpression group compared to the control group (Fig. 7b, c, Supplementary Table S13). Fibrosis activation markers (*Postn, Col3a1, Ltbp2)* were significantly inhibited in *Pknox2*-overexpressing cells (Fig. 7d). The pathway enrichment analysis revealed that downregulated genes were involved in extracellular matrix organization, mesenchymal cell differentiation, and collagen fibril organization, whereas upregulated genes were associated with cell proliferation (Fig. 7e, f). We performed independent assays and validated that *Pknox2* overexpression restrained TGFβ-induced fibrosis genes expression (*Col1a1* and *Postn*) and SMAD2 pathway activation (Fig. 7g–i). An analysis of published chromatin immunoprecipitation (ChIP) assays^40^ on murine tissue revealed that PKNOX2 can directly bind to downstream fibrogenic genes such as *Postn*, *Col1a1* and *Col5a1*, suggesting direct transcriptional regulation of fibrosis gene expression (Supplementary Fig. 9).

**Fig. 8:**
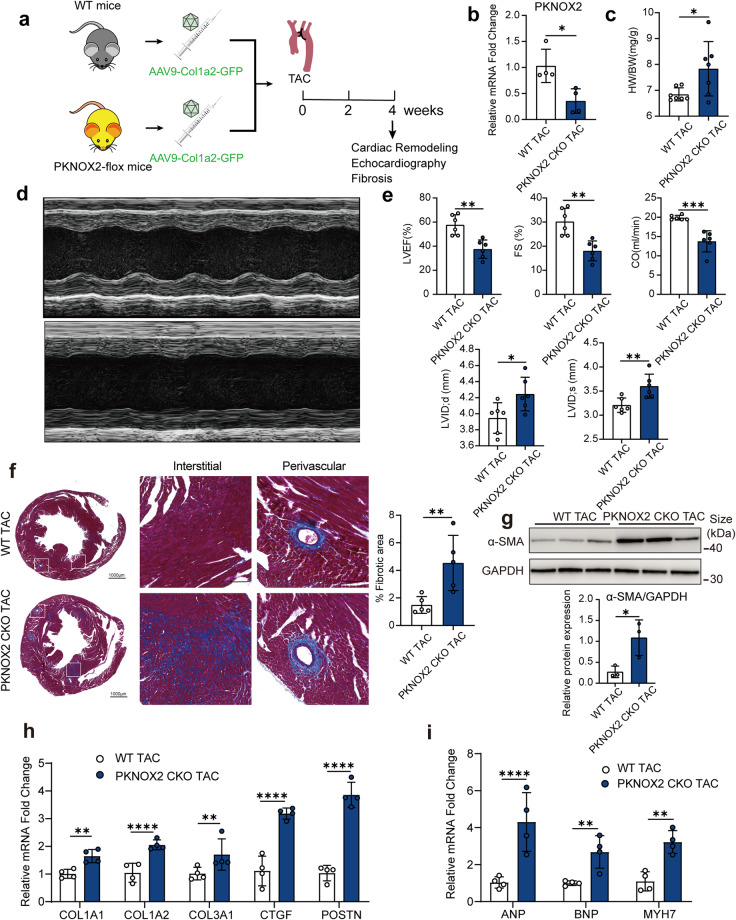
Pknox2 knockout exacerbates transverse aortic constriction (TAC)–induced cardiac fibrosis in mouse hearts. **a** Experiment design. **b** PKNOX2 expression level in the heart of WT and PKNOX2-CKO (*n* = 4 per group) mice is measured by real-time qPCR. **c** Heart weight (HW)/body weight (BW) ratios in WT TAC and PKNOX2-CKO mice at 4 weeks after TAC surgery (*n* = 7 per group). **d**, **e** Assessments of echocardiographic parameters of left ventricular ejection fraction (LVEF%), fraction shortening (FS%), cardiac output (CO ml/min), left ventricular end-systolic internal diameter (LVID;s), and left ventricular end-diastolic internal diameter (LVID;d) in PKNOX2-CKO and WT mice at 4 weeks after TAC surgery (*n* = 6 per group). **f** Representative images of Masson staining of LV cross-sections in the hearts of PKNOX2-CKO and WT mice at 4 weeks after 4 surgery (*n* = 5 per group). Scale bar, 100 μm. Quantitative results of LV interstitial collagen volume from the indicated groups (*n* = 5 per group). **g** Representative western blots and statistical analysis of α-SMA protein expression in the hearts of PKNOX2-CKO and WT mice at 4 weeks after TAC surgery (*n* = 3 per group). **h**, **i** Relative mRNA levels of fibrosis and heart failure marker genes in heart tissues from the indicated mice (*n* = 4 per group). All data are presented as mean ± SD. For statistics, one-way ANOVA with Bonferroni post hoc analysis was used. **P < 0.01, ***P < 0.001, ****P < 0.0001

To confirm the effect of PKNOX2 in pathological myocardial fibrosis, we generated fibroblast-specific PKNOX2 knockout mice (AAV9-Col1a2-Cre&PKNOX2 flox; Pknox2 CKO) and induced a heart failure model through transverse aortic constriction (TAC) operation (Fig. 8a). The successful conditional knock-out of PKNOX2 was confirmed by qPCR using heart samples (Fig. 8b). Four weeks after the TAC operation, the heart weight/body weight ratios of Pknox2 CKO mice were substantially higher than those of control mice (Fig. 8c). The in-vivo echocardiographic evaluation verified the reduced myocardial function and enlarged left ventricular chamber in Pknox2-CKO mice compared with controls (Fig. 8d, e). Pknox2-CKO mice also showed obviously exacerbated TAC-induced cardiac fibrosis, as shown by Masson staining (Fig. 8f). The expression of genes and proteins related to fibrosis and heart failure was also obviously upregulated in Pknox2-KO mice (Fig. 8g–i). Overall, loss-of-function of Pknox2 aggravates pressure overload–induced cardiac fibrosis and dysfunction.

**Fig. 9:**
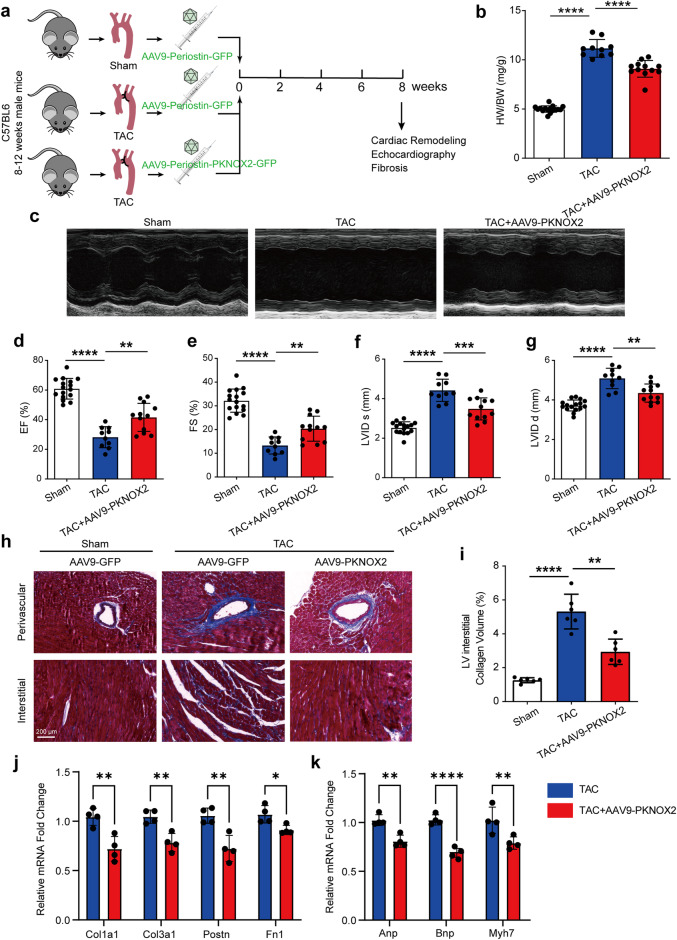
Specific overexpression of Pknox2 inhibits transverse aortic constriction (TAC)–induced cardiac fibrosis in mouse hearts. **a** The timeline of the experimental procedure in adult mice. **b** Heart weight (HW)/body weight (BW) ratios in adeno-associated virus serotype 9 (AAV9)-GFP (green fluorescent protein) and AAV9-Pknox2 mice at 4 weeks after sham or TAC surgery (*n* = 16, 10, 12). **c**–**g** Assessments of echocardiographic parameters of ejection fraction (EF%), fraction shortening (FS%), left ventricular end-systolic internal diameter (LVIDs), and left ventricular end-diastolic internal diameter (LVIDd) in AAV9-Periostin-GFP and AAV9-Postn-Pknox2-GFP mice at 8 weeks after sham or TAC surgery (*n* = 16, 10, 12). **h** Representative images of Masson staining of LV cross-sections in the hearts of AAV9-Periostin-GFP and AAV9-Postn-Pknox2-GFP mice at 8 weeks after sham or TAC surgery (*n* = 6 per group). Scale bar, 200 μm. **i** Quantitative results of LV interstitial collagen volume from the indicated groups (*n* = 6 per group). **j** Relative mRNA levels of fibrosis and heart failure marker genes in heart tissues from the indicated mice (*n* = 4). All data are presented as mean ± SD. For statistics, one-way ANOVA with Bonferroni post hoc analysis was used. **P < 0.01, ***P < 0.001, ****P < 0.0001

The fibroblast-specific AAV serotype 9–delivered *Pknox2* enhanced by the Periostin promoter (labelled as AAV9-Postn-Pknox2-GFP) was constructed to overexpress Pknox2 in vivo, while AAV9-Postn-GFP was used as a control (Fig. 9a). Overexpression specificity and efficiency were validated using immunofluorescence (Supplementary Fig. 10). Eight weeks after the TAC operation, the heart weight/body weight ratios of Pknox2-overexpressing mice were substantially lower than those of control mice (Fig. 9b). An echocardiographic evaluation revealed the effective improvement of myocardial function in the AAV9-Postn-Pknox2-GFP group compared with the controls after TAC (Fig. 9c–g). In pathology, fibroblast-specific overexpression of *Pknox2* obviously alleviated TAC-induced fibrosis, as shown by Masson staining (Fig. 9h, i). The expression of genes related to fibrosis and heart failure was also obviously downregulated in AAV9-Postn-Pknox2-GFP mice (Fig. 9j, k). Overall, these results indicated overexpression of Pknox2 alleviated pressure overload-induced cardiac fibrosis.

Collectively, these results demonstrated that PKNOX2 plays an essential inhibitory role in pathological myocardial fibrosis and might be a potential therapeutic target in myocardial remodelling during heart failure.

## Discussion

**Fig. 10:**
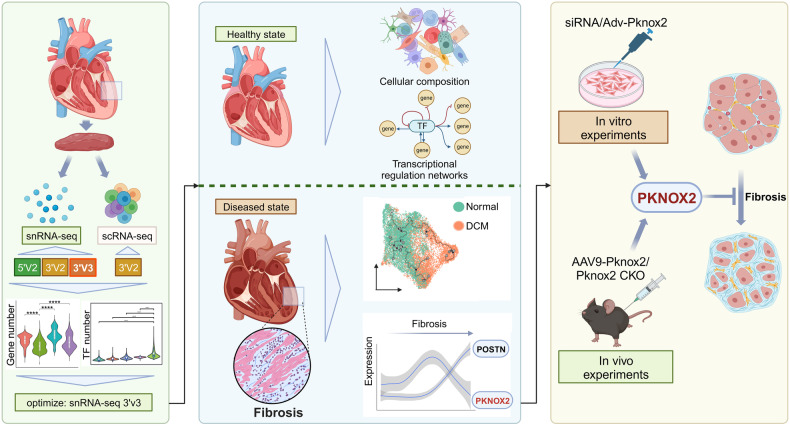
Research scheme. The workflow of the whole study. We developed a new tissue processing and single-nucleus transcriptomic profiling pipeline that improved the detection of more TFs and genes in single-nucleus RNA sequencing. With this optimized protocol, we compared the cellular composition and transcriptional regulation networks of healthy hearts and failing heart, in which we identified *PKNOX2* as an essential inhibitory regulator in myocardial fibrosis remodelling. In vitro and in vivo experiments further confirm our conclusion. sn, single nucleus; sc, single cell; TF, transcription factor; DCM, dilated cardiomyopathy; Adv, adenovirus. This figure was created with BioRender.com

Although a series of studies on the human cardiac cell atlas have been released in recent years,^5,9,11,13,41^ the transcriptional regulation landscapes of cardiac cells and their subtypes in the determination of the cell identities remain under study, as the low abundance of TFs increases the difficulty in detection at the single-cell level. Herein, we established a nuclear isolation protocol in combination with the snRNA-seq pipeline to achieve unbiased cellular profiling and higher detected numbers of TFs than in previous datasets. With this optimized pipeline, we successfully characterized the proportions of cell types in healthy human hearts and described the transcriptional regulation pathways in maintaining homeostasis of subpopulations of each cardiac cell type. Furthermore, to validate the essential roles of TFs in maintaining cellular homeostasis, a group of failing hearts was enrolled to study the dysregulation of TFs, with a focus on the activation of fibroblasts during myocardial remodelling. Among the TFs, *PKNOX2* was demonstrated to be a novel negative regulator of fibroblast activation and was validated by in vivo and in vitro experiments (Fig. 10).

Building a human cell atlas requires expression profiling of human tissues from different samples, including difficult-to-dissociate tissues, tissues composed of fragile cells, and frozen specimens, all of which are incompatible with scRNA-seq. As an alternative, snRNA-seq is increasingly used to analyse human tissue heterogeneity. However, the detection ability of genes by snRNA-seq was considered inferior to that of scRNA-seq, especially for low-abundance TFs. In the present study, a FACS-free protocol was developed to obtain nuclear suspensions from heart muscle with little debris and high purity. Therefore, our dataset compiled with the optimized protocol contained significantly higher numbers of detected genes and TFs than previously published datasets,^5,9,11,14^ strengthening the transcriptional regulation analysis. Moreover, our snRNA-seq results presented significantly lower numbers of mitochondrial and ribosomal RNAs than are in previously published datasets,^5,9,11,14^ suggesting less contamination by cytoplasmic RNA. It is worth noting that all snRNA-seq datasets consisted of data from frozen tissues, and therefore, a comparison of the performance of this method with fresh and frozen heart tissues has yet to be conducted. A similar quality estimation study indicated that both fresh dissociated and frozen cryosectioned kidney tissues retained sufficient nuclear RNA levels for snRNA-seq assays, and global gene and transcript levels appeared unaffected.^42^

On the other hand, it is unclear whether snRNA-seq can replace scRNA-seq across different cell types and tissues, although previous studies have compared Drop-seq and DroNc-seq in adult mouse kidneys.^43^ Herein, we performed the first head-to-head comparison of scRNA-seq and snRNA-seq on human heart tissues. This analysis allowed us to understand the similarities and differences between the two high-throughput technologies and to recognize the biases introduced by the sequencing technology. Given the large number of reads aligned to intronic regions by snRNA-seq,^42–44^ the inclusion of introns for analysis significantly improved the sensitivity of snRNA-seq, consistent with the findings of previous studies.^43,45,46^ With the optimized protocols and analysis pipelines, the number of detected genes in datasets compiled with snRNA-seq was comparable or even superior to that obtained by scRNA-seq. In terms of cell clustering, nuclear sequencing can be a good substitute for intact-cell profiling. However, a substantial number of genes was detected differentially between these two techniques, such as with scRNA-seq exhibiting a preference for immune-related antigens and mitochondrial oxidative phosphorylation genes, and snRNA-seq showing a preference for genes involved in signalling pathways. These novel observations can guide the choice of an appropriate strategy for single-cell profiling based on the study aims. It is particularly necessary to point out that most of the comparisons above were based on a single sample in each group, so the conclusion would be stronger if multiple runs were included in the comparisons in the future.

Together, in contrast to scRNA-seq and previously published datasets, our results optimized the snRNA-seq protocol, which generated good nuclei prep and performed better in detecting TFs and genes involved in signalling pathways and in deciphering transcriptional regulation landscapes of cardiac cells. Moreover, this FACS-free protocol also implies the potential for revisiting cellular composition in an unbiased manner, but more exact side-by-side comparisons need to be performed.

Although multiple research has focused on the cell proportions in the heart, but the results remain controversial, especially for nonmyocytes.^4,47,48^ Through transcriptome profiling to accurately define cell identity and unbiased flow sorting-independent nuclei isolation techniques that employ a physical dissociation strategy, the present study provided an unbiased and systemic quantification of cellular composition in the heart muscles at a rigorous resolution. The fractions of cardiomyocyte nuclei in the ventricles were approximately 30% in our datasets, consistent with previous reports.^4,15^ The cellular proportions differ from the anatomical regions of myocardium. The datasets compiled from our previous study revealed that the fractions of CM nuclei in atria were approximately 20%, lower than those in ventricles (~30%).^10^ These findings provide an update to the previously reported observation that different regions have similar CM proportions.^4^ Our data supported that fibroblasts compose the largest proportion of nonmyocytes, which is consistent with the findings of previous studies,^49^ whereas pericytes were identified at an unexpectedly high proportion (15%) that was much higher than the previously reported value of approximately 5%.^50^ The reason for the underestimation of pericytes and fibroblasts might be that gene signatures of these cells were not well recognized before single-cell/nucleus profiling was applied, and the traditional identification of cell identity by one or several known surface markers could not represent the whole pericyte and fibroblast population. The extremely close contact with ECs might limit the observation and quantification of pericytes by histological examinations. The unexpectedly high ratio of pericytes to ECs (1:1) might greatly contribute to endothelial barrier function and EC turnover,^16^ as well as oncological cardiotoxicity^51^ and myocardial repair.^52,53^ Our previous studies also indicated abundant ligand‒receptor interactions and coexpressed gene signatures along the vasculature between pericytes and ECs.^10^ This evidence suggests the need for further investigation into the function and clinical significance of pericytes. However, it must be mentioned that our results are a nuclear based cell composition limited to our detection methods. It may reveal cellular composition of the human heart at the nuclear level.

Owing to the high-resolution detection of genes involved in regulation signalling, our dataset enables/enabled the deciphering of transcriptional regulation networks that potentially govern the cell identities and homeostasis among the main cardiac cell subpopulations. Regarding the vascular cells whose gene signatures have been well described along the vasculature in our previous report,^10^ the present study further identified the TFs that were involved in regulating arteriole-capillary-venous vascular function. Among ECs, of which the functions and regulatory mechanisms have been most recognized,^54–56^ our study confirmed the vascular functions and transcriptional regulators of individual subpopulations, consistent with the findings of previous functional studies, such as regarding *PRDM16* being involved in angiogenesis and blood pressure regulation in artECs,^17,18^ *PPARG* being associated with lipid metabolism and EC proliferation in capECs, and *FOXP1* being responsible for cytokine binding and leucocyte adhesion in venECs.^24^ These results, in turn, validated the robustness and reliability of our analysis strategy. This study also identified novel TFs enriched in specific EC subsets that might be involved in vascular remodelling in myocardial diseases such as myocardial infarction and inflammatory heart disease. *SOX5* was enriched in artECs, possibly associated with arteriogenesis in the AMI model, as it has been demonstrated to induce angiogenesis in oncogenesis and osteogenesis.^57,58^ *ZBTB7C* enrichment in venECs is potentially responsible for vascular inflammation in the chronic ischaemic cardiomyopathy model. Interestingly, this gene has been reported to be associated with the stroke infarct size by a genome-wide association study,^59^ and our study may provide a potential explanation of the intrinsic mechanism. Regarding the pericytes that were under detected before, our study identified many TFs involved in cellular function maintenance. Alternative expression of these TFs was observed in myocardial infarction, but none of them has been studied before.

In the present study, we validated many regulators that have been demonstrated to play essential roles in maintaining vascular homeostasis, indicating the robustness and reliability of our analysis pipeline. Meanwhile, more novel regulators, especially in pericytes, were identified by our study, and the intrinsic mechanisms of the novel TFs in governing homeostasis and their contributions to myocardial injury and repair remain to be illuminated.

Fibroblasts are a group of heterogeneous cell subpopulations with high plasticity and flexible cell states.^60^ They constitute a prominent component of the myocardial microenvironment and participate in almost all myocardial diseases. Unlike ECs with distinct subtypes, the definitions of subclusters within fibroblasts differ across studies, an issue that is not well recognized.^5,11,13,41^ Based on the high-quality snRNA-seq data, we identified distinct cell subclusters with definitional functions and gene signatures for fibroblasts in healthy human hearts, which were successfully transferred to a previous human cell atlas and fibroblasts from diseased hearts.^5,11^

In contrast to previously published heart failure profiling studies,^11,13^ our healthy heart study identified the gene regulation networks responsible for early-stage fibroblast activation and the initiation of myocardial fibrosis rather than those responsible for advanced-stage myoFB differentiation. We identified the top first TF, *MEOX1*, which has recently been demonstrated to play a crucial role in fibroblast activation.^38^ In a previous study, *MEOX1* was identified by screening a reversible transcriptional switch that underlies fibroblast activation using single-cell chromatin accessibility analysis of hearts dynamically exposed to bromodomain and extraterminal domain (BET) inhibitors. Among the most dynamic DNA elements was an enhancer that regulated *MEOX1*, which was demonstrated to be needed for TGFβ-induced fibroblast activation by experimental validation.^38^ The top rank of *MEOX1* in our TF analysis on fibroblast activation also verified the reliability of our datasets and analysis pipelines, and *MEOX1* was labelled as a landmark of the fibroblast activation trajectory. Along with this trajectory, we identified a series of TFs that have been previously demonstrated to be involved in myocardial fibrosis, such as *RUNX1*^61^ and *AEBP1*.^7,62^

Interestingly, a novel regulator, *PKNOX2*, was identified, next only to *MEOX1* at the trajectory of fibroblast activation. It is worth mentioning that *PKNOX2* was not increased in bulk RNA profiling. Further analysis of fibroblasts from diseased hearts with DCM and series of validation experiments revealed that PKNOX2 increased in fibroblast activation but decreased in advanced fibrosis formation (myofibroblast induction) during myocardial remodelling. This dynamic switch of *PKNOX2* is different from what occurs in previously reported regulators involved in fibrosis activation. In contrast to what occurs with fibrogenic *MEOX1*, our study demonstrated that knockdown of Pknox2 induced activation of fibroblasts, similar to induction by TGFβ, whereas overexpression alleviated the fibrosis induced by TGFβ, suggesting a fibrosis inhibition potency of *PKNOX*2. The downstream signalling pathways were explored at cellular level, and we found that the classic TGFβ-SMAD3 pathway was activated, which may serve as a possible mechanism for *PKNOX2* regulated fibrosis activation and formation. In vitro experiments confirmed *PKNOX2* effects in pathological myocardial fibrosis, through generating *PKNOX2* overexpression and fibroblast-specific knockout mouse line, we found that *PKNOX2* knockout aggravated cardiac disfunction and exacerbated cardiac fibrosis, while *PKNOX2* overexpression had an opposite effect in TAC induced heart failure mice.

Chip-seq and ATAC-seq are also important approaches towards studying TFs in healthy and diseased state nowadays. We used the published Chip-seq dataset to confirm the PKNOX2 possible downstream regulatory pathways. The Chip-seq results also suggested that *PKNOX2* could directly bind to promoter of some fibrosis genes, such as *POSTN* and *COL1A1*, suggesting that it may directly play a transcriptional regulatory role.

Chromatin accessibility analysis is a widely used method to study transcriptional regulatory networks. The ChIP-seq results suggested that *PKNOX2* could directly bind to promoters of some fibrosis genes, such as *POSTN* and *COL1A1*, suggesting that it may directly play a transcriptional regulatory role. ATAC-seq is another approach to detecting the unique chromatin landscape and how it may be altered by perturbation or disease.^63,64^ In our future study, we will further elucidate the specific regulatory mechanism of PKNOX2 including its downstream genes and exact pathway through ATAC-seq. More importantly, PKNOX2 has been demonstrated to interact with MBD3,^65^ which is a component of the histone deacetylase NuRD complex that participates in the remodelling of chromatin.^66^ MBD3 is widely recognized as a transcriptional repressor and is involved in gene silencing through interaction with other DNA-binding proteins.^67,68^ These findings, together with our gain- and loss-of-function results, warrant speculation that PKNOX2 might interact with the NuRD complex and subsequently repress fibrogenic gene expression.

Moreover, the pathway enrichment analysis for the RNA-seq data revealed that PKNOX2 might regulate cell proliferation, suggesting multilevel regulation by *PKNOX2* in the pathogenesis of heart remodelling. The intrinsic mechanisms of *PKNOX2*, including transcriptional activation sites and exact target genes, remain to be further elucidated through cell chromatin accessibility analysis and chromatin immunoprecipitation assays. Nevertheless, the in vivo study using the murine TAC model demonstrated a convincing therapeutic effect of enhancing *PKNOX2* on alleviating myocardial fibrosis and cardiac dysfunction, indicating a novel potential antifibrotic target in heart failure remodelling. This representative regulator that was unchanged by bulk gene profiling again emphasized the strength of single-cell profiling strategies in studying cellular identities and modulation mechanisms in homeostasis and disease remodelling.

In summary, this study established an optimized nuclear isolation and snRNA-seq pipeline with unbiased nuclear selection and favourable sequencing quality. With this optimized pipeline, we characterized the cellular composition of healthy hearts and illuminated the transcriptional regulation networks in cell subpopulations of major cardiac cell types in the healthy state. Furthermore, to study the function of these TFs in maintaining homeostasis with a focus on myocardial fibrosis remodelling, a heart failure model was used in which we identified PKNOX2 as an essential regulator in myocardial fibrosis remodelling.

## Materials and methods

Detailed methods for all protocols used in this study were provided in the Expanded Materials and Methods in the Supplementary files.

### Human sample collection

Donor and diseased human hearts (DCM) were obtained from the transplantation center of Fuwai Hospital (Beijing, China). All donors were males and experienced brain death caused by haemorrhagic stroke or cerebral trauma from traffic accident and high-altitude falling during construction work. These hearts were selected from those that were initially considered for heart transplantation but were ultimately abandoned due to a size mismatch. The heart sample collection for pathology and molecular study was approved by the Medical Ethics Committee of Fuwai Hospital (Approval No: 2013-496), and the transplanted hearts harvested for cell isolation and culture study (cardiac cell biobank) was approved by the same committee (Approval No: 2017-887). Written informed consent with detailed research purpose of human material was obtained from each patient or their relatives. Information regarding human hearts utilized in this study is available in Supplementary Table S1.

### Single-nucleus and single-cell RNA-seq

The 10X genomics chromium platform (10X genomics, USA) was used to perform both single-nucleus and single-cell RNA-seq. In pipelines evaluation, we used three different Chromium Single Cell Reagents, 3’ V2, 5’ V2 and 3’ V3, separately, for library preparation according to the manufacturer’s instructions. In subsequent transcriptional regulation analysis, all datasets derived from healthy and diseased hearts were generated separately using the optimized protocol by Chromium Single Cell Reagents 3’V3.

### Experimental animals

All experiments involving animals were conducted in accordance with the Guide for the Use and Care of Laboratory Animals. All animal protocols were approved by the Institutional Animal Care and Use Committee (IACUC, approval number FW-2022-0051), Fuwai Hospital, Chinese Academy of Medical Sciences.

### Statistics

All values are presented as mean ± SD. Two-group comparisons were performed with an unpaired, 2-tailed Student’s *t* test or non-parametric statistics. Multiple group comparisons were performed with one-way ANOVA. Bonferroni adjustment was used for *p* value in multiple testing. A value of *p* < 0.05 was considered statistically significant. All statistical analyses were performed in R4.1.2.

### Supplementary information

Supplemental Material

Supplemental Table 1

Supplemental Table 2

Supplemental Table 3

Supplemental Table 4

Supplemental Table 5

Supplemental Table 6

Supplemental Table 7

Supplemental Table 8

Supplemental Table 9

Supplemental Table 10

Supplemental Table 11

Supplemental Table 12

Supplemental Table 13

Supplemental Table 14

Supplemental Table 15

Supplemental Table 16

## Data Availability

The data that support the findings of this study, including scRNA-seq/snRNA-seq data and R scripts for single-cell data analysis, are available online and from the corresponding author upon reasonable request. The analysis scripts were deposited on https://github.com/eleozzr/HeartReproduce. The data that support the findings of this study are available from Chen, Liang; Li, Haotong; Liu, Xiaorui; Zhang, Ningning; Wang, Kui; Shi, Anteng; et al. (2023). Massively Parallel Single-Nucleus RNA-Sequencing allows an Unbiased Assessment of Cellular Constituents of the Adult Human Heart and Transcriptional Regulators Involved in Myocardial Remodelling. figshare. Dataset. 10.6084/m9.figshare.23732883. All other data and information supporting the findings are available in the article and Supplementary files.

## References

[1] Murray CJ, Lopez AD (2013). Measuring the global burden of disease. N. Engl. J. Med..

[2] GBD 2016 Causes of Death Collaborators. (2017). Global, regional, and national age-sex specific mortality for 264 causes of death, 1980-2016: a systematic analysis for the Global Burden of Disease Study 2016. Lancet.

[3] Roth GA (2015). Demographic and epidemiologic drivers of global cardiovascular mortality. N. Engl. J. Med..

[4] Pinto AR (2016). Revisiting cardiac cellular composition. Circ. Res..

[5] Litvinukova M (2020). Cells of the adult human heart. Nature.

[6] Asp M (2019). A spatiotemporal organ-wide gene expression and cell atlas of the developing human heart. Cell.

[7] Rao M (2021). Resolving the intertwining of inflammation and fibrosis in human heart failure at single-cell level. Basic Res. Cardiol..

[8] Chen Z, Wei L, Duru F, Chen L (2020). Single-cell RNA sequencing: in-depth decoding of heart biology and cardiovascular diseases. Curr. Genomics.

[9] Tucker NR (2020). Transcriptional and cellular diversity of the human heart. Circulation.

[10] Chen L (2022). Multifaceted spatial and functional zonation of cardiac cells in adult human heart. Circulation.

[11] Reichart D (2022). Pathogenic variants damage cell composition and single cell transcription in cardiomyopathies. Science.

[12] Nicin L (2022). A human cell atlas of the pressure-induced hypertrophic heart. Nat. Cardiovasc. Res..

[13] Chaffin M (2022). Single-nucleus profiling of human dilated and hypertrophic cardiomyopathy. Nature.

[14] Cui M (2020). Dynamic transcriptional responses to injury of regenerative and non-regenerative cardiomyocytes revealed by single-nucleus RNA sequencing. Dev. Cell.

[15] Bergmann O (2015). Dynamics of cell generation and turnover in the human heart. Cell.

[16] Nees S (2012). Isolation, bulk cultivation, and characterization of coronary microvascular pericytes: the second most frequent myocardial cell type in vitro. Am. J. Physiol. Heart Circ. Physiol..

[17] Matrone G (2021). Fli1(+) cells transcriptional analysis reveals an Lmo2-Prdm16 axis in angiogenesis. Proc. Natl Acad. Sci. USA.

[18] Craps S (2021). Prdm16 supports arterial flow recovery by maintaining endothelial function. Circ. Res..

[19] Mouillesseaux KP (2016). Notch regulates BMP responsiveness and lateral branching in vessel networks via SMAD6. Nat. Commun..

[20] Takeda N (2004). Endothelial PAS domain protein 1 gene promotes angiogenesis through the transactivation of both vascular endothelial growth factor and its receptor, Flt-1. Circ. Res..

[21] Zhou M (2021). Roquin2 suppresses breast cancer progression by inhibiting tumor angiogenesis via selectively destabilizing proangiogenic factors mRNA. Int J. Biol. Sci..

[22] McCracken IR (2022). Mapping the developing human cardiac endothelium at single cell resolution identifies MECOM as a regulator of arteriovenous gene expression. Cardiovasc. Res..

[23] Rosano S, Parab S, Noghero A, Cora D, Bussolino F (2022). Long non-coding RNA LINC02802 regulates in vitro sprouting angiogenesis by sponging microRNA-486-5p. Int. J. Mol. Sci..

[24] Zhuang T (2019). Endothelial Foxp1 suppresses atherosclerosis via modulation of Nlrp3 inflammasome activation. Circ. Res..

[25] Ruiz-Villalba A (2020). Single-cell RNA sequencing analysis reveals a crucial role for CTHRC1 (Collagen Triple Helix Repeat Containing 1) cardiac fibroblasts after myocardial infarction. Circulation.

[26] van Duijvenboden K (2019). Conserved NPPB+ border zone switches from MEF2- to AP-1-driven gene program. Circulation.

[27] Meng Z (2023). Cationic proteins from eosinophils bind bone morphogenetic protein receptors promoting vascular calcification and atherogenesis. Eur. Heart J..

[28] Federti E (2017). Peroxiredoxin-2 plays a pivotal role as multimodal cytoprotector in the early phase of pulmonary hypertension. Free Radic. Biol. Med..

[29] Bonta PI (2010). Nuclear receptor Nurr1 is expressed in and is associated with human restenosis and inhibits vascular lesion formation in mice involving inhibition of smooth muscle cell proliferation and inflammation. Circulation.

[30] Schupp JC (2021). Integrated single-cell atlas of endothelial cells of the human lung. Circulation.

[31] da Silva RMS, de Mello RJV (2017). Fat deposition in the left ventricle: descriptive and observational study in autopsy. Lipids Health Dis..

[32] Yang XZ (2018). LINC01133 as ceRNA inhibits gastric cancer progression by sponging miR-106a-3p to regulate APC expression and the Wnt/beta-catenin pathway. Mol. Cancer.

[33] Tokue M (2017). SHISA6 confers resistance to differentiation-promoting wnt/beta-catenin signaling in mouse spermatogenic stem cells. Stem Cell Rep..

[34] Doucet-Beaupre H, Ang SL, Levesque M (2015). Cell fate determination, neuronal maintenance and disease state: the emerging role of transcription factors Lmx1a and Lmx1b. FEBS Lett..

[35] Pearson RC, Funnell AP, Crossley M (2011). The mammalian zinc finger transcription factor Kruppel-like factor 3 (KLF3/BKLF). IUBMB Life.

[36] Ilsley MD (2017). Kruppel-like factors compete for promoters and enhancers to fine-tune transcription. Nucleic Acids Res..

[37] Nakamura Y (2020). A disintegrin and metalloproteinase 12 prevents heart failure by regulating cardiac hypertrophy and fibrosis. Am. J. Physiol. Heart Circ. Physiol..

[38] Alexanian M (2021). A transcriptional switch governs fibroblast activation in heart disease. Nature.

[39] Heymans S, Lakdawala NK, Tschöpe C, Klingel K (2023). Dilated cardiomyopathy: causes, mechanisms, and current and future treatment approaches. Lancet.

[40] Penkov D (2013). Analysis of the DNA-binding profile and function of TALE homeoproteins reveals their specialization and specific interactions with Hox genes/proteins. Cell Rep..

[41] Koenig AL (2022). Single-cell transcriptomics reveals cell-type-specific diversification in human heart failure. Nat. Cardiovasc. Res..

[42] Lake BB (2019). A single-nucleus RNA-sequencing pipeline to decipher the molecular anatomy and pathophysiology of human kidneys. Nat. Commun..

[43] Wu H, Kirita Y, Donnelly EL, Humphreys BD (2019). Advantages of single-nucleus over single-cell RNA sequencing of adult kidney: rare cell types and novel cell states revealed in fibrosis. J. Am. Soc. Nephrol..

[44] Habib N (2017). Massively parallel single-nucleus RNA-seq with DroNc-seq. Nat. Methods.

[45] Lake BB (2017). A comparative strategy for single-nucleus and single-cell transcriptomes confirms accuracy in predicted cell-type expression from nuclear RNA. Sci. Rep..

[46] Mereu E (2020). Benchmarking single-cell RNA-sequencing protocols for cell atlas projects. Nat. Biotechnol..

[47] Zhou P, Pu WT (2016). Recounting cardiac cellular composition. Circ. Res..

[48] O’Farrell FM, Attwell D (2014). A role for pericytes in coronary no-reflow. Nat. Rev. Cardiol..

[49] Nag AC (1980). Study of non-muscle cells of the adult mammalian heart: a fine structural analysis and distribution. Cytobios.

[50] Lee LL, Chintalgattu V (2019). Pericytes in the Heart. Adv. Exp. Med Biol..

[51] Chintalgattu V (2013). Coronary microvascular pericytes are the cellular target of sunitinib malate-induced cardiotoxicity. Sci. Transl. Med..

[52] Chen CW (2013). Human pericytes for ischemic heart repair. Stem Cells.

[53] Katare R (2011). Transplantation of human pericyte progenitor cells improves the repair of infarcted heart through activation of an angiogenic program involving micro-RNA-132. Circ. Res..

[54] Aird WC (2007). Phenotypic heterogeneity of the endothelium: I. Structure, function, and mechanisms. Circ. Res..

[55] Aird WC (2007). Phenotypic heterogeneity of the endothelium: II. Representative vascular beds. Circ. Res..

[56] Dejana E, Hirschi KK, Simons M (2017). The molecular basis of endothelial cell plasticity. Nat. Commun..

[57] Chen X (2018). SOX5 induces lung adenocarcinoma angiogenesis by inducing the expression of VEGF through STAT3 signaling. Onco Targets Ther..

[58] Fang S (2022). Pro-angiognetic and pro-osteogenic effects of human umbilical cord mesenchymal stem cell-derived exosomal miR-21-5p in osteonecrosis of the femoral head. Cell Death Discov..

[59] Du R (2015). Integrative mouse and human studies implicate ANGPT1 and ZBTB7C as susceptibility genes to ischemic injury. Stroke.

[60] Tallquist MD, Molkentin JD (2017). Redefining the identity of cardiac fibroblasts. Nat. Rev. Cardiol..

[61] Riddell A (2020). RUNX1: an emerging therapeutic target for cardiovascular disease. Cardiovasc Res..

[62] Liu X (2023). Lineage-specific regulatory changes in hypertrophic cardiomyopathy unraveled by single-nucleus RNA-seq and spatial transcriptomics. Cell Discov..

[63] Grandi FC, Modi H, Kampman L, Corces MR (2022). Chromatin accessibility profiling by ATAC-seq. Nat. Protoc..

[64] Ranzoni AM (2021). Integrative single-cell RNA-Seq and ATAC-Seq analysis of human developmental hematopoiesis. Cell Stem Cell.

[65] Luck K (2020). A reference map of the human binary protein interactome. Nature.

[66] Hoffmeister H (2017). CHD3 and CHD4 form distinct NuRD complexes with different yet overlapping functionality. Nucleic Acids Res..

[67] Morey L (2008). MBD3, a component of the NuRD complex, facilitates chromatin alteration and deposition of epigenetic marks. Mol. Cell Biol..

[68] Saito M, Ishikawa F (2002). The mCpG-binding domain of human MBD3 does not bind to mCpG but interacts with NuRD/Mi2 components HDAC1 and MTA2. J. Biol. Chem..

