# Supplementary material for: PBX/Knotted 1 homeobox-2 (PKNOX2) is a novel regulator of myocardial fibrosis
Source: Signal Transduct Target Ther. 2024 Apr 22;9:94. doi: 10.1038/s41392-024-01804-5 (PMC11033280; doi:10.1038/s41392-024-01804-5)
Supplement: Supplementary file 1 — Supplemental Material [file 41392_2024_1804_MOESM1_ESM.docx]

**Supplementary Materials for**

***PBX/Knotted 1 homeobox-2（PKNOX2）is a novel regulator of myocardial fibrosis***

Liang Chen, Haotong Li, Xiaorui Liu, Ningning Zhang, Kui Wang, Anteng Shi, Hang Gao, Deniz Akdis, Ardan M. Saguner, Xinjie Xu, Elena Osto, Willem Van de Veen, Guangyu Li, Antoni Bayés-Genís, Firat Duru, Jiangping Song, Xiangjie Li, Shengshou Hu

Correspondence to: huss@fuwaihospital.org

**This PDF file includes:**

Materials and Methods

Figures. S1 to S10

Captions for Tables S1 to S16

**Materials and Methods**

**Sample collection**

Each heart was perfused with ice-cold University of Wisconsin (UW) solution (Bridge to Life, USA) simultaneously with dissection from the chest and shipped to the laboratory on ice within 4 hours of cold ischaemia time. Fresh tissue was sampled immediately for cell dissociation and single-cell RNA-seq. The LV myocardium was then dissected and immediately frozen in liquid nitrogen for single-nucleus RNA-seq. In order to obtain the hearts of animals, all animals were sacrificed under anaesthesia with 2% isoflurane, and the LV myocardium of the heart was immediately dissected, followed by washing in ice-cold PBS buffer to remove the blood and flash freezing in liquid nitrogen. The samples used in each study design were shown in **Table S1**.

**Nucleus isolation and quality control**

All nuclei isolation procedures were conducted on ice or at 4°C, and RNase inhibitor (RI, 80 U/ml) (Invitrogen, USA) was added to all buffers to protect the RNA from degradation. The frozen heart tissue (300 mg) was thawed on ice and cut into pieces at 1 mm^2^ size, and washed in ice-cold PBS buffer once. The tissues pieces were then transferred to a 50 ml Falcon tube containing 10 ml of lysis buffer (32 M sucrose, 5 mM CaCl_2_, 3 mM C_4_H_6_MgO_4_, 0.5 mM EGTA, 10 mM Tris-HCl 8.0, 2 mM EDTA, 1 mM PMSF, 1 mM DTT, and 80U/ml RI). Then, the tissue suspension was homogenized with a T-25 Ultra-Turrax probe (IKA, Germany) at 24,000 rpm for 15 seconds. Another 20 ml of lysis buffer was added to generate a 30 ml suspension in a Falcon tube. This suspension was further homogenized with 10 strokes using a glass Dounce homogenizer with a tight pestle, followed by incubation on ice for 10 minutes to lyse the cells. The crude nucleus suspension was consecutively passed through 100 µm and 70 μm nylon mesh cell strainers (Corning, USA) and then centrifuged at 700 × g for 10 minutes at 4°C. The supernatant was carefully removed, and the crude nucleus isolate was resuspended in 30 ml sucrose buffer (2.1 M sucrose, 3 mM C_4_H_6_MgO_4_, 10 mM Tris-HCl 8.0, 1 mM PMSF, 1 mM DTT, and 80 U/ml RI). Then, the crude nucleus suspension in sucrose buffer was slowly overlaid on the added 10 ml sucrose buffer in an ultracentrifuge tube to avoid disturbing the interface, followed by centrifugation at 20,000 g for 1 h at 4°C (Beckman Avanti S-25). The supernatant was carefully removed, and 1 ml of nucleus suspension buffer (1% BSA, 200 U/ml RI in PBS) was used to dissolve the nuclear pellet in an ultracentrifuge tube. The nucleus suspension was transferred to a 15 ml Falcon tube and supplemented with 9 ml of nucleus suspension buffer to wash the nuclei. The nucleus suspension was passed through a 30 μm nylon mesh cell strainer (Miltenyi Biotec, Germany) and then centrifuged at 500 × g for 10 minutes at 4°C. The nuclear pellet was then resuspended in 1 ml of nucleus suspension buffer and subjected to further experiments.

A portion of the nucleus suspension was used to extract total nuclear RNA using TRIzol. The total nuclear RNA was then analysed with an Agilent 2100 Bioanalyzer to evaluate RNA integrity on the basis of the abundance of 18S and 28S rRNA and the RIN value. This quality control was used to assess the efficiency of RNA protection during nuclear isolation, and nuclear RNA integrity directly influences the quality of snRNA-seq results.

In this protocol, the nuclei were isolated by mechanical separation without enzyme digestion or flow soring, thus the nuclei from all cell types were obtained by this protocol.

**RNA integrity evaluation**

Before nucleus isolation, total RNA was extracted from a small piece of the prepared frozen heart tissue with TRIzol (Invitrogen, USA), according to the manufacturer’s protocols. Then, the RNA was separated via electrophoresis with an Agilent 2100 Bioanalyzer (Agilent Technologies, USA) to evaluate RNA integrity on the basis of the abundance of 18S and 28S rRNA and the RNA integrity number (RIN) value. A sample was selected for single-nucleus RNA-seq when the RIN value was higher than 8.0.

**Cell dissociation and sorting**

Cells were dissociated from fresh human heart tissue using a neonatal heart dissociation kit (Miltenyi Biotec, Germany, 130-098-373) with gentleMACS equipment (Miltenyi Biotec) according to the manufacturer’s instructions. This protocol could destroy adult cardiomyocytes and isolate mainly the nonmyocytes in adult myocardial tissue, as we reported previously^1^. Briefly, the harvested fresh heart tissue was cut into small pieces (1-2 mm^3^) and transferred into a gentleMACS C tube with an enzyme mix consisting of enzymes P, A, and D. The sample was incubated without agitation for 15 minutes at 37°C and then attached to a gentleMACS Dissociator with the gentleMACS Program mr_neoheart_01. Then, the sample was resuspended in 7.5 ml of cell culture medium with FBS (Gibco, USA) to terminate the program and passed through a cell strainer (70 μm), followed by washing once with cell culture medium containing FBS. The cell suspension was then subjected to red blood cell lysis using Red Blood Cell Lysis Solution (Miltenyi Biotec, Germany). After washing one time, the resuspended cells were prepared for further study. Flow cytometry was used to remove cell debris and sort viable cells with 7-amino-actinomycin D (7-AAD, Invitrogen 00-6993-50) staining. The obtained cell suspension was subjected to scRNA-seq. In this protocol, most cardiomyocytes were damaged, so we mainly obtained nonmyocytes in scRNA-seq.

**Single-nucleus and single-cell RNA-seq**

The 10X genomics chromium platform (10X genomics, USA) was used to perform both single-nucleus and single-cell RNA-seq. The nucleus suspension was quantified with a cell counter (Countstar BioTech, China) and diluted to 800~1200 nuclei/µl. In total, 16,000 nuclei were subjected to a droplet-based platform for single-nucleus selection for a targeted capture of 8,000~10,000 nuclei in each run. We used three different Chromium Single Cell Reagents, 3’ V2, 5’ V2, and 3’ V3, separately, for library preparation according to the manufacturer’s instructions. The mRNA underwent reverse transcription and PCR preamplification, with 15 cycles for the 3’ V2/5’ V2 kit and 14 cycles for the 3’ V3 reagent. The PCR product (cDNA) obtained after preamplification was separated via electrophoresis using an Agilent 2100 Bioanalyzer to evaluate cDNA quality. The cDNA was further subjected to complete library construction. We finally used a HiSeq X10 (Illumina, USA) for sequencing after quality control of the cDNA library. For the single-cell RNA-seq, we used Chromium Single Cell Reagent 3’ V2 to sequence cell suspension isolated by enzyme digestion, as described in the previous section. The detailed protocol was similar to the single-nucleus experiment as described before, according to the manufacturer’s instructions.

**Bioinformatics analysis**

*Data preprocessing*

10X sequencing data were analysed by using Cell Ranger Single-Cell Software (version 3.0.2, 10X Genomics) with the hg38 human reference genome(https://cf.10xgenomics.com/supp/cell-exp/refdata-cellranger-GRCh38-3.0.0.tar.gz) for each sample. As suggested by the Cell Ranger website, the snRNA-seq assay captures both unspliced pre-mRNA and mature mRNA, so we created a custom “pre-mRNA” reference package listing each gene transcript locus (including the intron genome) as an exon reference. Thus, these intronic reads were included in the UMI counts for each barcode.

To obtain high-quality cells, we first filtered out barcodes (cells) with <500 UMI counts, <200 expressed genes, or >5% of mitochondrial genes. To remove doublets, we used three different methods (doubletfinder^2^, scrublet^3^, and doubletdection^4^), and the cells identified by at least two algorithms were removed as doublets.

*Dimension reduction and cell type identification*

Based on the above preprocessing approach, we conducted clustering of the remaining cells. Gene expression values (or UMI count matrix) for each cell were normalized to count-per-ten-thousand, log-transformed with the *NormalizeData* function in Seurat v3^5^. A set of highly variable genes was identified by *FindVariableFeatures with* parameter *nfeatures=2000* for each sample. After running ScaleDate and RunPCA in Seurat, we used FindNeigbors (with top 20 PCs) and *FindClusters* (with npcs=20) to identify clusters*.* Then, cell identity was assigned by manual annotation using known marker genes and computed differentially expressed genes (DEGs). The DEGs for each cluster were identified for at least 10% of the cells in either of the two populations compared, and the fold change in expression was at least exp (0.6). Then, cell type was assigned by manual annotation using known marker genes (**Table S14**).

*Combination analysis, batch effect correction and cell clustering for multiple healthy samples*

We first normalized the raw gene expression values (or UMI count matrix) for each cell were normalized to count-per-ten-thousand, log-transformed with the *NormalizeData* function in Seurat v3^5^. We used the standard pipeline of Seurat for integrating and analyzing single-cell data. In detail, we firstly extract the top 3000 highly variable genes for each sample, and then we used the shared highly variable gene as the *anchor.feature* for *FindIntegrationAnchors* function with the parameter dims=1:30 and k.filter=200 in Seurat, and then we integrated data using the *IntegrateData* function with dims=1:30. The combined data was scaled using the ScaleData function. Principal component analysis (PCA) is performed using the RunPCA function. An elbow plot was generated to determine the optimal number of principal components to retain in the subsequent analysis. Using the optimal number of principal components, we used the integrated data to find neighbors (FindNeighbors with k.parm=20) and perform clustering analysis (FindClusters with resolution=0.5). Dimensionality reduction techniques, TSNE (t-distributed stochastic neighbor embedding) or Uniform Manifold Approximation and Projection (UMAP), were applied to the integrated data.

*Data integration, batch effect correction and cell clustering for multiple samples from diseased hearts*

In the snRNA-seq analysis of heart failure samples, we enrolled three healthy hearts and three transplanted hearts with DCM. The datasets were generated separately, and combined together for downstream analysis. We firstly normalized the raw gene expression values (or UMI count matrix) for each cell to count-per-ten-thousand, log-transformed with the *NormalizeData* function in Seurat v3^5^. A set of highly variable genes was identified by *FindVariableFeatures with* parameter *nfeatures=2000* for each sample. After running ScaleDate and RunPCA in Seurat, the ideal principal components (PCs) were calculated and corrected by different donors with Harmony to regress out the variation introduced by different donors^6^. “Harmony”-corrected PC 1-50 were used to construct a K nearest neighbor (KNN) graph with K= 30 and to identify unsupervised cell clusters. Resolutions ranging from 0.1 to 0.5 were chosen for different cell cluster identifications. For dimension reduction, a Uniform Manifold Approximation and Projection (UMAP) was implemented on 1-50 “Harmony” embedding to visualize cell clustering.

*Subclustering analysis for cardiac cells*

For subcluster analysis of ECs, fibroblasts, pericytes and SMCs from healthy heart, we used the standard pipeline of Seurat for cell integrating and clustering as the previous procedures of “*Combination analysis, batch effect correction and cell clustering for multiple healthy samples*” section. And we used the resolution 0.2, 0.4, 0.2, 0.4 for subtype identification of ECs, fibroblasts, pericytes and SMCs, respectively.

For fibroblast analysis from diseased hearts with DCM, we firstly preprocessed with the standard pipeline from R package monocle3. And then we utilized Monocle3's functionalities to align samples, reduce dimensionality and cluster cells (with resolution=8e-4), facilitating accurate characterization of cell sub-populations.

*Gene scores evaluation*

Enrichment scores were calculated using the Seurat function *AddModuleScore*. The *AddModuleScore* function calculated the average expression of a gene set by subtracting the aggregated expression of control gene sets, which could be deemed the average relative expression.

*Differential expression analysis*

Differential expression (DE) tests for a gene between a pair of clusters or between a cluster and the remaining clusters were performed using the *FindAllMarker* function in Seurat (with the default test method: Wilcoxon rank sum test; p values were adjusted for multiple testing using the Bonferroni correction). The numbers of detected UMIs and expressed genes were compared.

*Transcription factor regulon analysis*

We applied single-cell regulatory network inference and clustering (SCENIC)^20^ analysis to identify the regulatory network and regulon activity of transcription factors (TFs) using the Python module pySCENIC (0.11.2). After running pySCENIC, we obtained a continuous and binary area under the curve (AUC) score for each regulation network, where the AUC value represents the regulon activity within the cell cluster. Differential regulons were identified by the Wilcoxon rank-sum test, and the obtained p values were subjected to correction with Benjamini–Hochberg multiple testing. The scaled expression of regulon activity was used to generate a heatmap.

*Pseudotime analysis*

Pseudotime analysis of fibroblast cells in healthy hearts was performed using the R package Monocle v2.22.0^7^. Data were log-normalized and cell-ordered based on DDRTree reduction of highly variable genes. The heatmap in Figure 5F was generated by using the “plot_genes_branched_heatmap” function in Monocle. Gene expression was smoothed into 100 bins along pseudospace using a nature spline with 3 d.f. (degrees of freedom). Genes contributing or related to pseudotime were identified by a few functions in the R package tradeseq and Monocle. We filtered out those genes with <10 expressed cells and an adjusted p-value >0.01. For trajectory analysis of fibroblasts in heart failure, monocle3 package^8^ was used to infer the pseudotime, and the alterations in gene expression along the trajectories were calculated accordingly.

*Gene ontology enrichment analysis*

Overrepresentation of GO terms in gene lists was evaluated using the *enrichgo* function in the R package *clusterProfiler* or *Metascape (http://metascape.org/)*. Gene symbols were first converted to EntrezIDs by the *bitr* function in *clusterProfile*. We showed only the top significant GO term (sort p value in increasing order and limit adjusted p value <0.05) unless otherwise stated.

*TransferData analysis*

In order to compare the consistency of cell clustering between two datasets, we conduct a projection of reference data onto a query object. After finding anchors, we use the TransferData() function to classify the query cells based on reference data (a vector of reference cell type labels). Because we have the original label annotations for scRNA-seq, we can evaluate how well our predicted cell type annotations match the full reference.

*PAGA connectivity analysis*

Partition-based graph abstraction (PAGA) provides an interpretable graph-like map of the arising data manifold^9^, based on estimating the connectivity of manifold partitions. This connectivity can be considered indicative of similarity to a certain extent. Therefore, we can use low-dimensional representations obtained from any batch correction method to construct a graph, and then utilize the PAGA algorithm to calculate the similarity between different cell types or groups. Specifically, we utilized the "sc.tl.paga" function from the Python package Scanpy to implement this functionality."

*Collection and analysis of published datasets*

To compare our results obtained by optimized protocol with previous datasets, we collected 4 published datasets (Tucker, Circulations, 2020^10^; Litvinukova, Nature, 2020^11^; Cui, Dev Cell, 2021^12^ and Reichart, Science, 2022^13^) and compared the number of expressed genes, number of UMI counts, percentage of transcripts mapping to mitochondrial genes, percentage of transcripts mapping to ribosome genes, and the number of detected TFs. The non-paired t-test with Bonferroni adjustment was used to compare the significance of various datasets.

**Flow cytometry**

In order to analyse the purity of nuclei, the nucleus suspension was stained with Hoechst at a dilution of 1:200 on ice in the dark for 30 min. Stained single-nucleus suspensions were washed with sorting buffer, and FACS sorting was performed on a FACSAria II cell sorter (BD Biosciences). In order to analyse the proportion of cardiomyocyte nuclei, the nucleus suspension was stained with an anti-human PCM-1 antibody (#HPA023370, Sigma, USA), followed by an Alexa Fluor® 488-conjugated secondary antibody. Stained single-nucleus suspensions were washed with sorting buffer, followed by analysis on a BioRad S3 Cell Sorter (BioRad, USA).

**Immunofluorescence**

The collected human LV heart tissue of donors, arrhythmogenic cardiomyopathy (ACM) and dilated cardiomyopathy (DCM) patients were fixed in 4% paraformaldehyde for 72 hours at room temperature, then gradient dehydrated in ethanol and xylene, and embedded in paraffin. 5 μm paraffin sections were prepared for immunofluorescence staining. After deparaffinizing and rehydrating, the sections underwent antigen retrieval with Tris-EDTA buffer (pH=9.0). Then, they were blocked with goat serum and 0.3% Triton X-100 (Sigma-Aldrich, USA) for 1 h at room temperature. Primary antibodies were incubated at 4⁰C overnight. After washing 3 times with PBST and 2 times with PBS, the slides were incubated with fluorescently labeled secondary antibodies (1:400) (Invitrogen, USA) and kept in the dark for 1 h at room temperature. Subsequently, slides were washed three times with PBST，twice with PBS, and then Mounting Medium with DAPI. The primary antibodies used in this study were listed as follows. To validate the cellular composition of healthy hearts revealed by snRNA-seq, we performed in-situ Immunofluorescence assay to quantify the proportions of cell with positive expression of cell-specific marker genes. These makers were selected according to recognized knowledge and the snRNA-seq analysis with relatively high specificities (>75) for identification of target cells, including PCM-1 (Sigma, SAB1306530, Rabbit. Marker of cardiomyocytes), DCN (abcam, ab175404, Rabbit. Marker of fibroblasts), PECAM1 (Abcam, ab9498, Mouse. Marker of ECs), PDGFRβ (Abcam, ab91066, Rat. Marker of pericytes), CD45 (abcam, ab10558, Rabbit. Marker of immune cels) and MYH11 (abcam, ab125884, Rabbit. Marker of SMCs). In addition, other antibodies used for examination of gene expression and distribution in this study included CD31(CST, D8V9, Rabbit), SOX5(Abcam, ab94396, Rabbit), PKNOX2 (Abcam, ab221616, Rabbit), Vimentin (Abcam, ab8978, Mouse), GLIS3 (Invitrogen, PA5-41677, Rabbit), ABCC9 (Novus Biologicals, NBP2-22403, Mouse), α-SMA (Abcam, ab124964, Rabbit), and POSTN (abcam, ab14041, Rabbit), GFP (Abcam, ab5449, Goat), Col1a1(Abcam, ab6308, Mouse), Rabbit IgG (Abcam, ab6702, Goat), Mouse IgG (Abcam, ab150113, Goat), cTnT (Abcam, ab209813, Rabbit). The immunofluorescence images were captured by confocal microscopy (LSM780, Zeiss, Germany) under the same screening criteria.

**Quantitation of cellular proportions by fluorescence immunohistochemistry**

Ten formalin-fixed, paraffin-embedded myocardial sections from five heart ventricles (two sections from each heart) were collected for quantitation of each cell type. The expression and localization of each cell marker selected from snRNA-seq were examined separately by a 7-Color Opal™ Manual IHC Kit (PerkinElmer, USA) according to the manufacturer’s instructions. The images were collected with a PerkinElmer Scanner (PerkinElmer, USA) and analysed with InForm software (PerkinElmer, USA). In the automatic imaging process, cardiac cells were identified and counted by DAPI through automatic segmentation. The positive staining cells were estimated by supervised machine learning, followed by automatic quantitation (**Supplementary Fig. 4**). The immune-staining and imaging process was performed by two separate technicians who were blinded to the cellular proportions observed from the snRNA-seq analysis.

**Cell isolation and culture, knock-down and overexpression of *Pknox2***

Adult mouse hearts (8-10 weeks, male) were dissociated by the Langendorff method as previously described **^14^**, and adult mouse cardiac fibroblasts were obtained by CD90.2 magnetic bead (Miltenyi Biotec, Germany) sorting. To immortalize fibroblasts, adult mouse cardiac fibroblasts were infected with SV40 T antigen expressing lentiviral particles**^15^**. Human cardiac fibroblasts were obtained by using the Neonatal Heart Dissociation Kit (Miltenyi Biotec, Germany) as the instruction described. Fibroblasts were then purified by the differential attachment characteristics of the cells. For cell culture, 0.1% gelatin (Sigma-Aldrich, USA) was used to plate the petri dish for 2 hours earlier. Cells were plated and cultured in Advanced DMEM/F-12 (Gibco, USA) + 1% Glutamax (Gibco, USA) + 2% FBS (Gibco, USA) medium at 37°C in 5% CO2.

At 60-70% cell confluence, siRNA (siPknox2, designed and produced by GenePharma, China) was transfected with Lipofectamine 3000 Reagent (Invitrogen, USA) and Optimal-MEM (Gibco, USA) according to the manufacturer’s protocols. The medium was changed after 6 h to avoid the toxicity of transfection reagents. 24 h after the addition of the transfection reagent, the medium was changed to the basal medium as described above with 0.5% FBS, then 10 ng/mL rmTGF-β (R&D, USA) was added to stimulate fibroblast activation. 24 h after TGF-β stimulation, cells were collected for further analysis. At the end of the experiment, knockdown efficiency was detected by RT-qPCR. The siRNAs for the experiment were listed in **Table S15**.

For Pknox2 overexpression, adenovirus was purchased from Vigene Biosciences (Jinan, China). Adenovirus (Ad) of Pknox2 (Ad-Pknox2-GFP-3Flag) was used for overexpressing Pknox2 and Ad-GFP-3Flag was used as a control. The immortalized mouse cardiac fibroblasts were infected with the corresponding adenoviruses at a multiplicity of infection (MOI) of 100 for 24 hours. Viewing green fluorescence under confocal microscopy can judge the efficiency of adenovirus infection. Then, the cells were treated with 5 ng/mL rmTGF-β (R&D, USA) for 12 hours to induce fibroblast activation. 12 h after TGF-β stimulation, cells were collected for further analysis including qPCR and immunofluorescence assay. The overexpression efficiency of Pknox2 was detected by RT-qPCR.

**RNA isolation, bulk RNA-seq, and RT-qPCR**

Total RNAs were isolated from the cultured cells by TRIzol (Invitrogen, USA) reagent according to the manufacturer’s instruction. 1000 ng total RNAs were reverse transcribed into cDNA with PrimerScript RT Master Mix (Perfect Real Time) (Takara, RR036A, Japan). Half of the cDNA was for bulk RNA-seq, and the other half was for RT-qPCR validation. Bulk RNA-seq was done by Novogene with their standard process, in which libraries were sequenced using the Illumina NovaSeq 6000 Sequencing System and a 150PE strategy. Genes with an average count of less than 1 were filtered out. DEGs were analyzed by limma R package. Adjust *p* value < 0.05 and |fold change| > 1.2 were considered as the differential expressed criteria. The volcano plots and the heatmaps were used to visualize DEGs by ggpubr and ComplexHeatmap R package. Enrichment analysis was performed by using the clusterProfiler R package to determine the biological functions and pathways of the DEGs. Enrichment terms with p-value < 0.05 and q-value < 0.05 were considered as statistical significant. PowerUp SYBR Green Master Mix (Applied Biosystems, USA) was used for quantitative PCR on Applied Biosystems QuantStudio 5 Real-Time PCR Detection System. The relative mRNA fold change was calculated by the 2^^-△△CT^ method. Primer sequences of Mus musculus are listed in **Table S16**.

**Western blot analysis**

The cells and heart tissue were lysed in a RIPA buffer (150 mM NaCl, 1% Triton X-100, 0.5% sodium deoxycholate, 0.1% SDS, 50 mM Tris pH8.0) that contained protease inhibitors (Roche, #04693132001) and PhosSTOP phosphatase inhibitor (Roche, #4906837001). After centrifugation (15000 g, 10 min, 4℃), cell lysate concentrations were determined by BCA Protein Assay (Beyotime institute of Biotechnology, #P0012). A total of 20 μg protein lysates were electrophoresed and separated using a 4%-12% SDS-PAGE (NuPAGE 4-12%, Bis-Tris, Invitrogen, #NP0322BOX,) and then transferred onto PVDF membranes (iBlot Transfer Stacks, Invitrogen, #IB401001). The membranes were then blocked with 5% Albumin Bovine V (Roche, #10735078001) at 25°C for 1-2 hours and then incubated over night at 4°C with the following primary antibodies diluted in primary antibody dilution buffer (Beyotime, #P0023A): PKNOX2(Abcam, #ab169458); α-SMA (CST, #19245); SMAD2 (CST, #5339); p-SMAD2 (CST, #3108); GAPDH (CST, #2118). Afterwards the membranes were incubated for 1 hour at 25 °C with HRP-conjugated secondary antibodies (Goat anti-Rabbit HRP-conjugated secondary antibody, ZSGB-BIO, #ZB2301; Goat anti-Mouse HRP-conjugated secondary antibody, ZSGB-BIO, #ZB2305. 1:5000). The antigen–antibody complexes were detected using a SuperSignal ECL kit (Thermo, #A38555) in a Western blotting detection system (FluroChem M, Protein Sample, USA). Results were expressed as density values normalized to GAPDH levels.

**Myocardial infarction mouse model**

Adult 8-10-weeks old male C57BL/6J mice were anesthetized by intraperitoneal administration of tribromoethanol (400 mg/kg) and ventilated with a rodent respirator. Then, the left anterior descending coronary artery was permanently occluded using an 8-0 polypropylene suture. The occlusion was confirmed by blanching of the anterior wall of the left ventricle and ST-segment elevation on the electrocardiogram. The chest was then closed with a 4-0 suture. For the sham group, mice underwent the same operation where the ligature of the left anterior descending coronary artery was not tied. Mice recovered from anesthesia under warm conditions with normal ventilation. After surgery, cardiac function was assessed by echocardiography using a VisualSonics Vevo 2100 ultrasound system (VisualSonics, Inc.) with a 40-MHz 550 probe transducer at 2 days post-operation. M-mode images and real-time 2D B-mode cine loops of short-axis views of the LV were acquired for cardiac function assessment. The quality and reproducibility of the MI model were assessed by ejection fraction (EF%). Mice with EF%<40% were included as successful models. The observer performing echocardiography analyses was blinded to the mouse model surgeon.

**Transverse aortic constriction mouse model**

Cardiac fibrosis and remodelling are induced by transverse aortic constriction (TAC) surgery as previously described ^16^. In brief, 8 to 12-week-old male mice were anesthetized with tribromoethanol (175 mg/kg, T48402, Sigma-Aldrich), and the efficiency of anesthesia was confirmed by the disappearance of toe contractile reflex. The left chest was opened, and the transverse aortic was ligated transversely with a 7-0 silk thread and a 26-gauge needle. After the needle removed, the chest was closed. We used Doppler echocardiographic analysis (VisualSonics, Vevo 2100) to confirm the complete contraction of the aorta. Mice in the sham operation group underwent identical operations in parallel without blocking the transverse aorta. Mice from both TAC and sham groups were subjected to the gene therapy in the following.

**AAV9 mediated knockout of *Pknox2* in fibroblasts *in-vivo***

AAV9 carrying Cre recombinase under the regulation of the fibroblast binding Col1a2 promoter (AAV9-Col1a2-Cre) were purchased from Vigene Biosciences Inc (Shandong, China). AAV9-Col1a2-Cre was diluted to 5 times by PBS, and then injected into adult mice by a 1 ml insulin syringe with a 30G needle via tail vein. Injection dose: AAV9-Col1a2-Cre 1.5×10^11^ v.g./mouse. The AAV9 was injected to the PKNOX2 flox/flox mice and control mice. One week after the injection, TAC surgery was performed (n=10 for PKNOX2 flox/flox group; n=10 for control group). Four weeks post TAC operation, cardiac function and left ventricular size were measured by echocardiography, and myocardial fibrosis was measured by Masson staining of paraffin-embedded sections of hearts.

**AAV9 mediated overexpression of *Pknox2 in-vivo***

Adeno-associated virus9 (AAV9) of Pknox2 overexpression (AAV9-Postn-Pknox2-GFP, AAV9-Pknox2) and AAV9 of control (AAV9-Postn-GFP, AAV9-Control) were purchased from Vigene Biosciences Inc. (Shandong, China). AAV9 of Postn-Pknox2-GFP, in which the fibroblast-specific *Periostin* promoter drives expression of GFP-tagged, was constructed to activate Pknox2 expression. AAV9-Postn-GFP was used as a control. AAV9-Pknox2 or AAV9-Control solution was diluted to 5 times by PBS, and then injected into adult mice by a 1 ml insulin syringe with a 30G needle via tail vein. Injection dose: AAV9-Pknox2 1.5×10^11^ v.g./mouse, AAV9-Control 1×10^11^ v.g./mouse. The AAV9 was injected *in vivo* 2 hours after the TAC model completion by tail vein injection (n=12 for AAV9-Pknox2 group; n=10 for AAV9-Control group). Eight weeks post TAC operation, cardiac function and left ventricular size were measured by echocardiography, and myocardial fibrosis was measured by Masson staining of paraffin-embedded sections from hearts.

**Echocardiography**

Eight weeks after TAC operations, heart function was measured by echocardiography as previously described ^17^. In detail, Cardiac function was assessed by echocardiography using a VisualSonics Vevo 2100 ultrasound system (VisualSonics, Inc.) and a 40-MHz 550 probe transducer. Animals were anesthetized with 1.0% isofluorane and hair was removed over the measurement area. The mice were then placed in a supine position on a 37℃ heating pad. The real-time 2D B-mode cine loops and M-mode images of short-axis views of the LV were acquired for cardiac structure and function assessment. Ejection fraction (EF%), fraction shortening (FS%), left ventricular end-systolic internal diameter (LVIDd) and left ventricular end-diastolic internal diameter (LVIDs) were measured at the papillary muscle level.

**Masson staining and quantitation of fibrosis**

The preparation of paraffin-embedded sections was as previously described. Masson staining of myocardial sections was carried out according to the manufacturer's instructions (Sigma, HT15). The images were obtained using Axio Scan.Z1, and fibrotic areas were quantified by ImageJ (IBM, USA). All operations and subsequent analyses were performed in a blinded fashion.

**Figure. S1.**

**
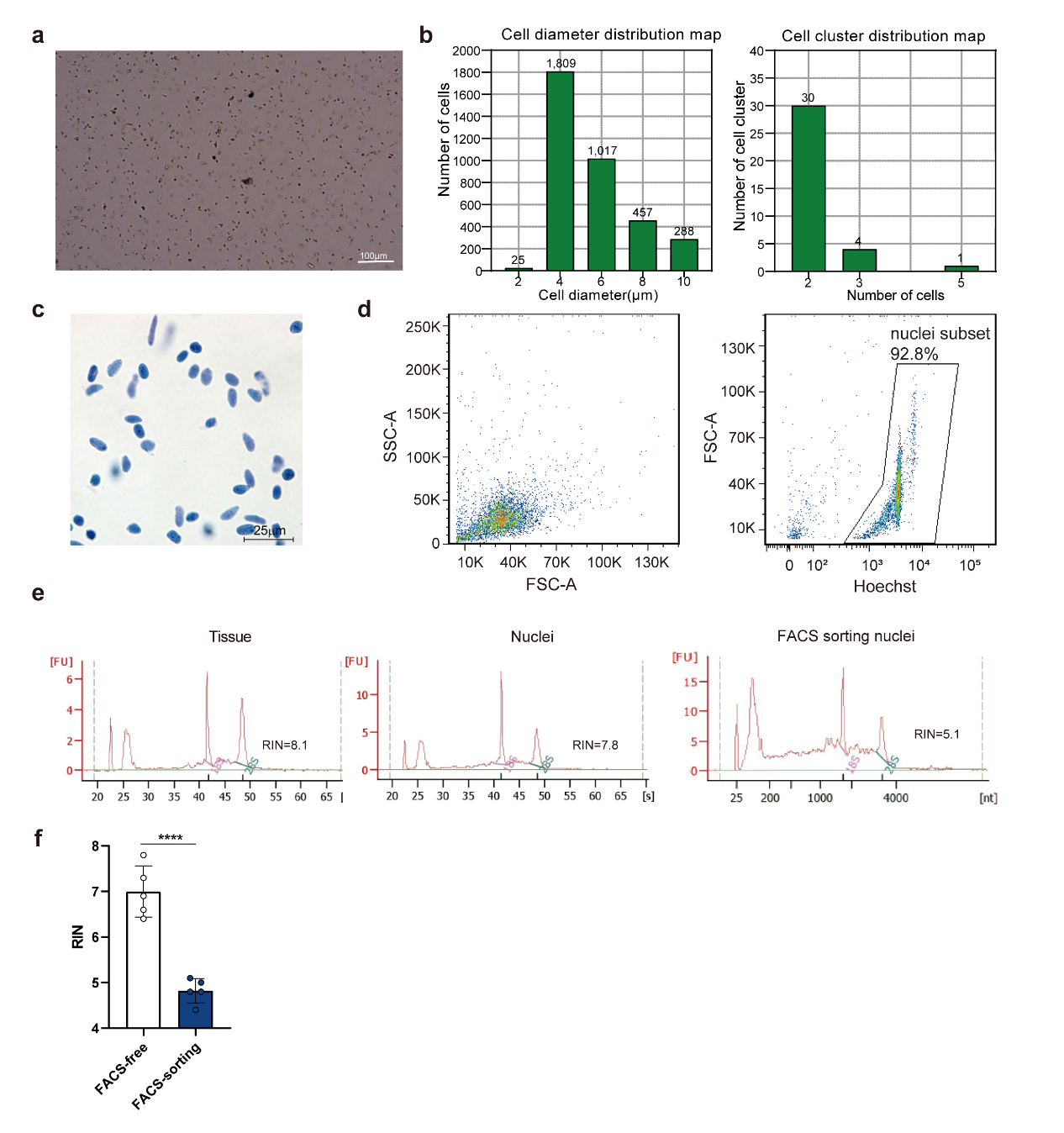
**

**Figure. S1. SnRNA-seq of human heart cells with RNA quality control.** (**a**) Quality control of nuclei by the light microscope. (**b**) Diameter distribution and cluster distribution of nuclei by Countstar. (**c**) Quality control of nuclei; Hoechst stained blue. The nucleus is blunt and round, the nuclear membrane is intact, and there is no aggregation, indicating that the quality of the nucleus is good. (**d**) The purity of nuclei was detected by flow cytometry with Hoechst staining, which indicated little cellular debris. (**e**) RNA integrity number (RIN) assessed by Agilent 2100 Bioanalyzer of RNA before (tissue) and after nuclei isolation by our optimized protocol and FACS sorting, respectively. (**f**) Nuclei suspension RIN of nuclei isolation by our optimized FACS-free protocol and traditional FACS-sorting methods. Data are represented as mean±SD. ****P < 0.0001.

**Figure. S2.**


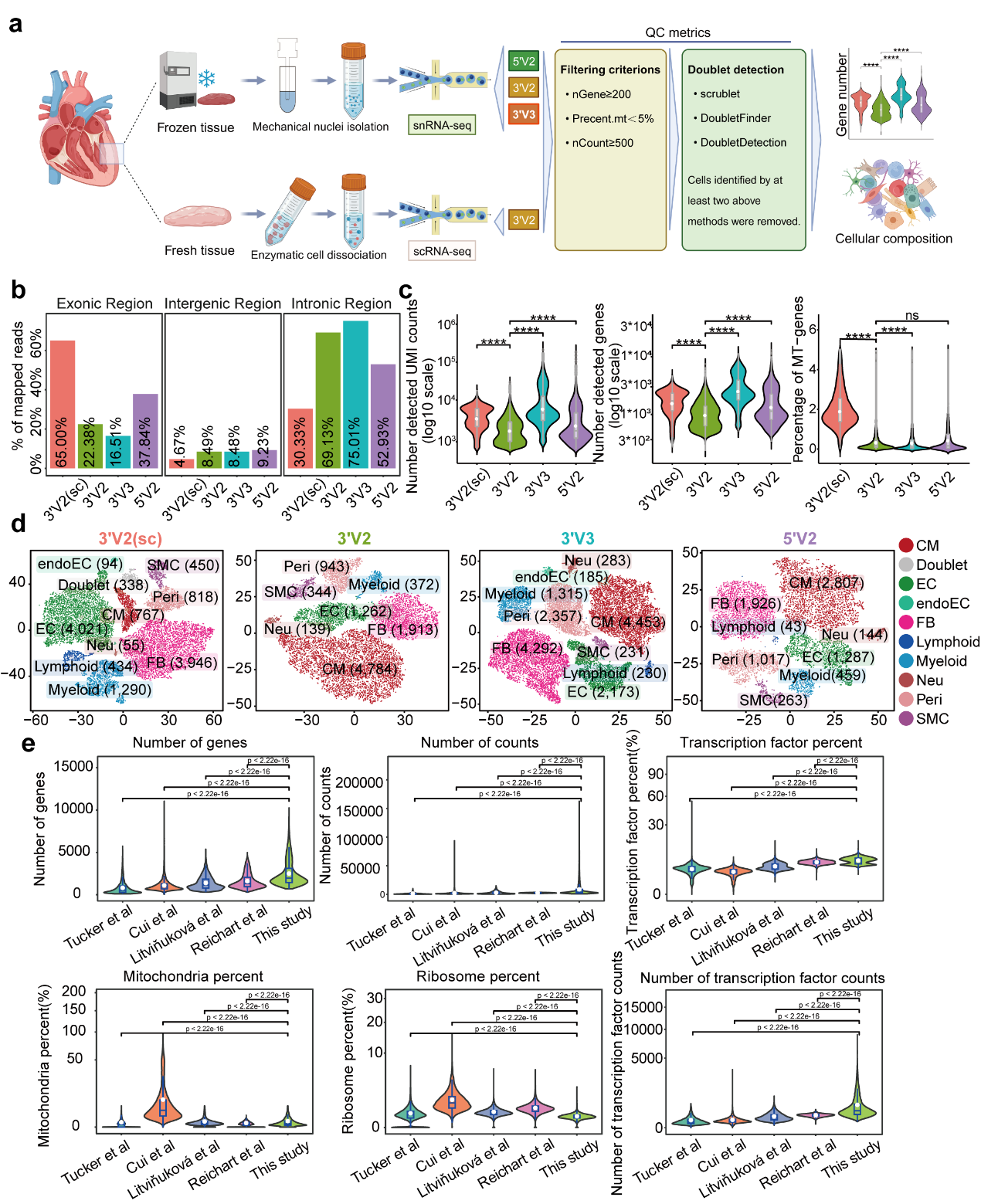


**Figure. S2. ScRNA-seq and snRNA-seq of human heart cells with different pipelines.** (**a**) The workflow for quality evaluation of four pipelines by single-nucleus/cell RNA sequencing on healthy human heart tissue. Four different library preparation kits were used: 5'V2, 3'V2, and 3'V3 for snRNA-seq (n=1 per group shown in Table S1) and 3'V2 for scRNA-seq (n=1 shown in Table S1). 5' and 3' kits capture and amplify RNA fragments from the 5' and 3' ends of mRNA, respectively. All datasets obtained by four pipelines were filtered with the same quality control (QC) metrics. (**b**) Reads mapped to exonic, intronic, and intergenic regions according to the protocol. (**c**) The median number of detected counts (left panel), genes (middle panel), and the average percentage of mitochondrial reads per cell across platforms and based on exonic reads only (only for single-cell RNA sequencing) or exonic and intronic reads together. (**d**) TSNE plots show the distribution of cell types for different protocols. CM, cardiomyocyte; EC, endothelial cell; endoEC, endocardial cell; FB, fibroblast; Neu, neutrophil; Peri, pericyte; SMC, smooth muscle cell. (**e**) Comparing the number of genes, number of counts, percentage of transcription factor, percentage of mitochondrial genes, percentage of ribosome genes, and number of transcription factor genes between our results and previously published four datasets. N=5 in each group. sn, single nucleus; sc, single cell. ***P < 0.001, ****P<0.0001.

**Figure. S3.**


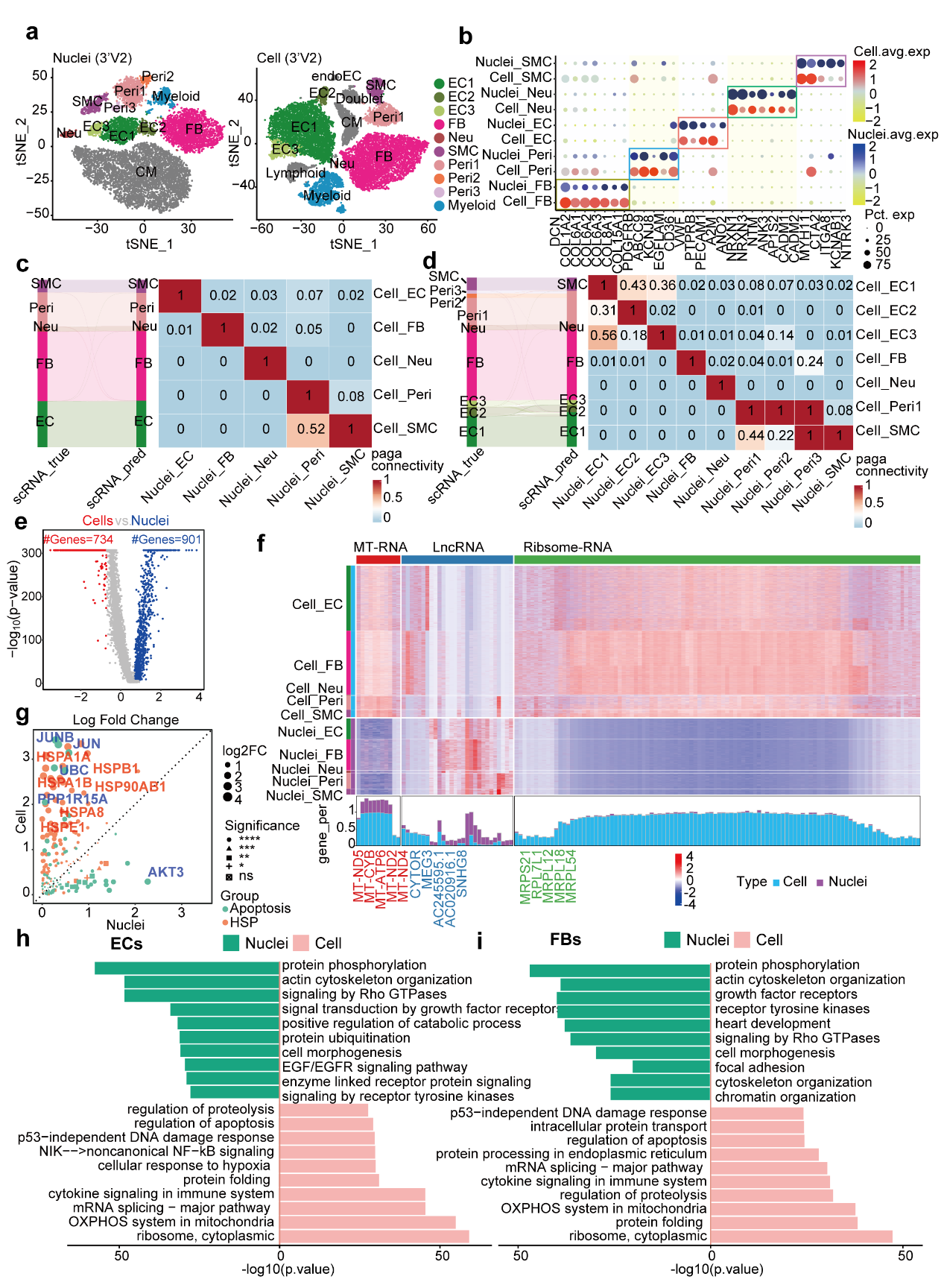


**Figure. S3. Comparison between scRNA-seq and snRNA-seq.** (**a**) TSNE plot showing the distribution of cell types for snRNA-seq and scRNA-seq. (**b**) Dot plot showing the classical marker genes of FBs, ECs, Peris, Neus and SMCs in scRNA-seq, and snRNA-seq. (**c-d**) Taking the gene expression profile of snRNA-seq as the reference, we used the TransferData function in Seurat3.0 to predict the consistency of cell types (**c**) and subtypes (**d**) for scRNA-seq. The left side of the Sankey plot shows the cell type labels obtained by scRNA-seq, and the right side of the Sankey plot shows the labels predicted by Seurat3.0 (left panel). The right panel shows the correlations between cell types obtained from scRNA-seq and snRNA-seq; the rows represent cells, and the columns represent nuclei. (**e**) Volcano plot showing the differentially expressed genes (DEGs) between scRNA-seq and snRNA-seq, with avg_logFC>=0.5, p_val_adj<0.01. (**f**) The heatmap showing the differential detection of mitochondrial gene (MT-RNA), long non-coding RNA (LncRNA), and ribosome RNA in scRNA-seq and snRNA-seq. (**g**) Scatter plots showing the difference in expression for stress-stimulated genes between scRNA-seq and snRNA-seq. Each point represents one gene. The x-axis indicates the average expression level in snRNA-seq, and the y-axis indicates the average expression level in scRNA-seq. The red bubbles represent heat shock protein genes, and the green bubbles represent apoptosis-related genes. The shape represented the significance between techniques. (**h-i**) Pathway enrichment analysis of endothelial cells (**h**) and fibroblasts (**i**) with DEGs between scRNA-seq and snRNA-seq which avg_logFC>=0.5, p_val_adj<0.01. HSP: heat shock protein, log2FC: log2 fold change.

**Figure. S4.**


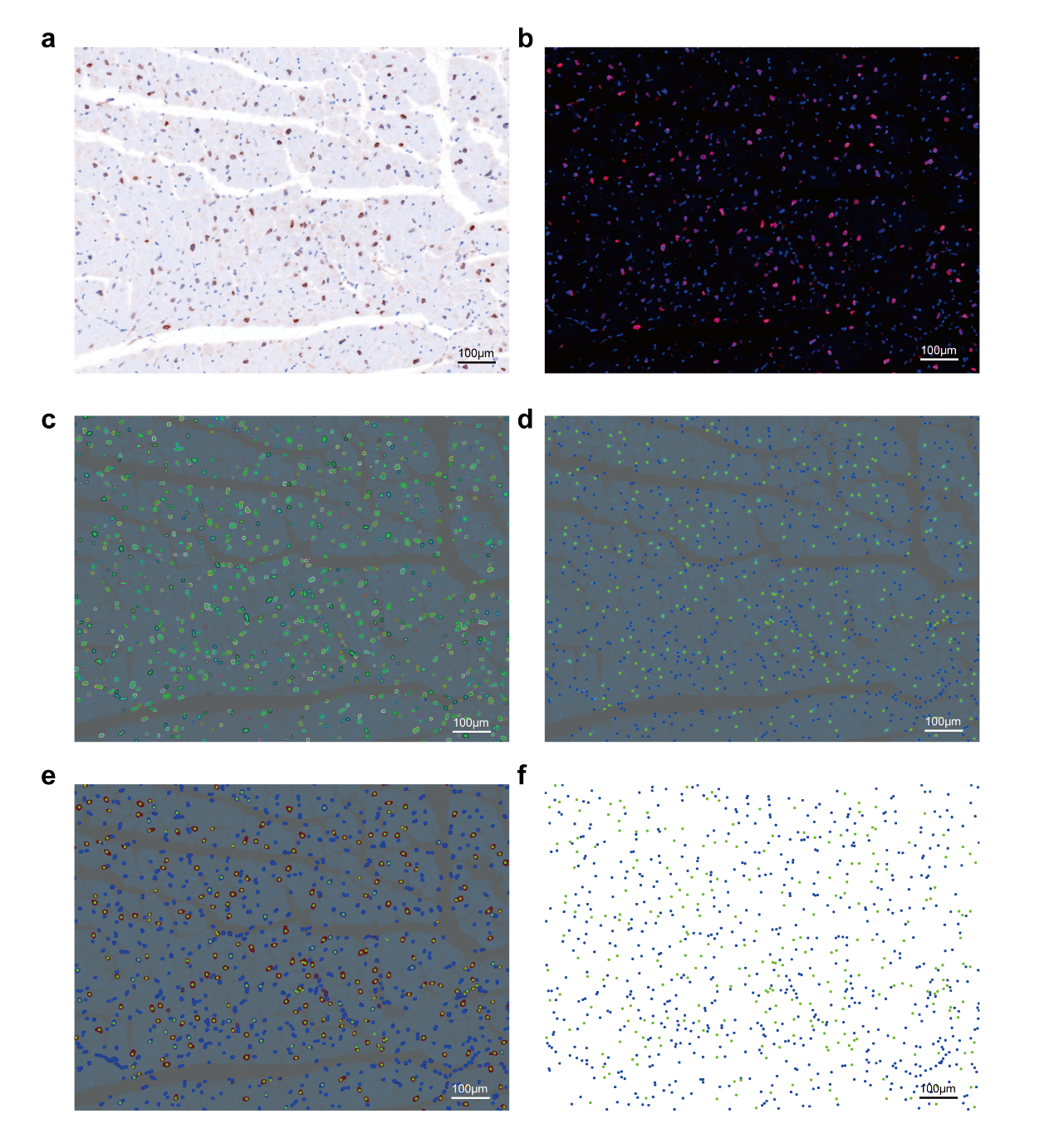


**Figure. S4. Analytic process of quantification of positive cells for fluorescence immunohistochemistry imaging.** (**a**) The original immunohistochemistry pathology image of PCM-1 staining. (**b**) The composite fluorescence image. (**c**) Automatic segmentation of nuclei. (**d**) The image with cell phenotype. The PCM-1 positive expression nuclei were labelled as green dots. (**e**) The combined image with cell phenotype (positive in green) and nuclei segmentation. (**f**) The purified image for counting nuclei in PCM-1 positive (green) and negative (blue) phenotype. All these images process was completed using InForm software (PerkinElmer, USA).

**Figure. S5.**


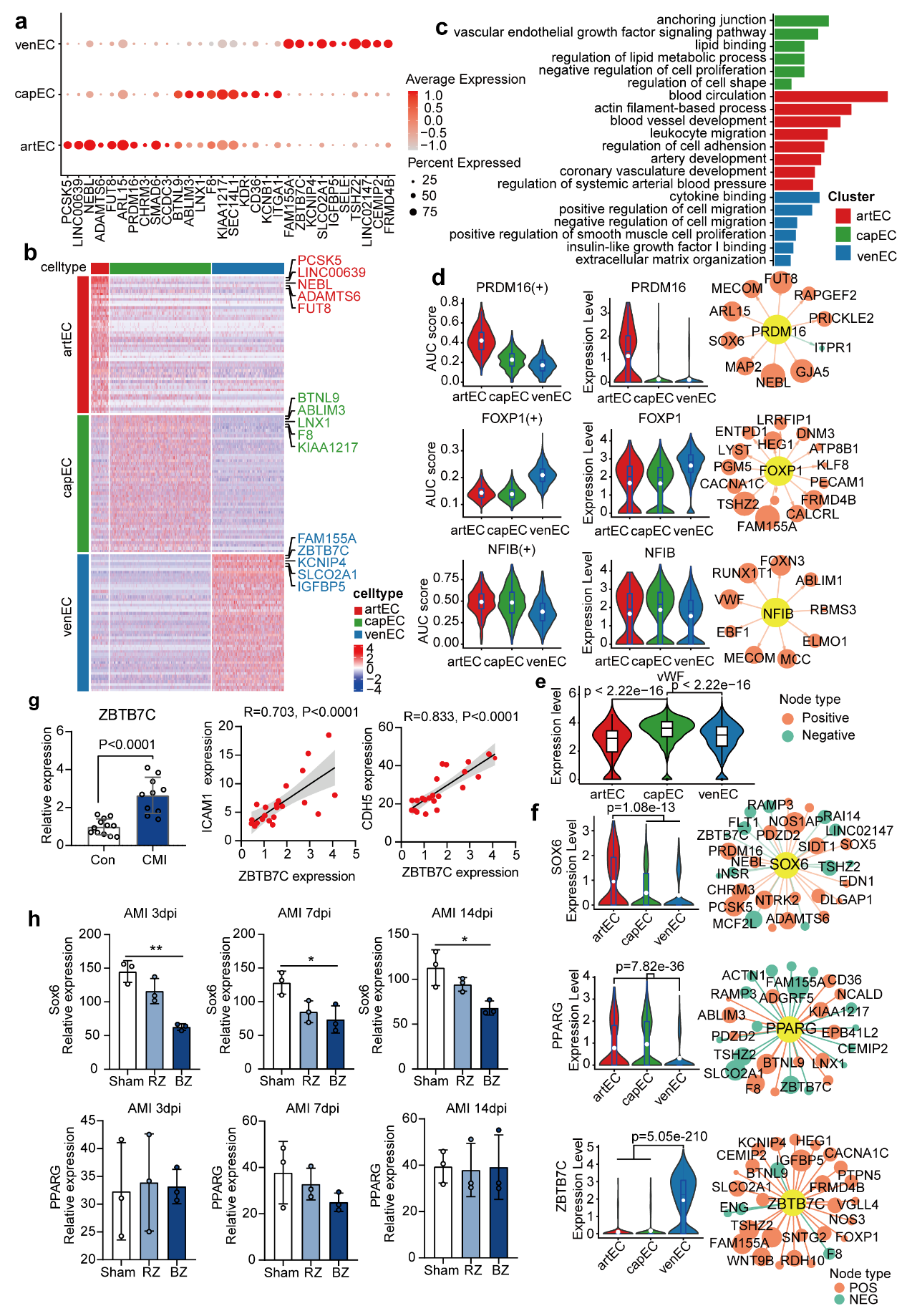


**Figure. S5. Endothelial cell subpopulations and gene regulation analysis.**

**a**) Marker genes of EC subpopulation. (**b**) Heatmap showing the highly expressed gene signatures of each EC subtype. (**c**) GO enrichment analysis of each EC subtype. (**d**) The regulon activities (evaluated by AUC score) and expression of representative TFs in artECs (*PRDM16*), venECs (*FOXP1*)and capECs (*NFIB*) and their regulatory networks. (**e**) Violin plot shows expression level of vWF in capEC higher than in artEC and venEC. (**f**) The expression levels and target gene networks of representative novel TFs in each vascular EC subtype (SOX6, PPARG, ZBTB7C). (**g**) Validation of *ZBTB7C* expression in the published bulk transcriptome dataset (GSE132146) of human explanted hearts with chronic myocardial infarction. Pearson correlation of *ZBTB7C* and the vascular lymphocyte adhesion molecules *ICAM1* and *CDH5*. (**h**) Validation of *Sox6* and *Pparg* expression in the bulk transcriptome dataset (GSE110209) of AMI murine model which includes remote zone and border zone at 3, 7, 14 dpi.

**Figure. S6.**


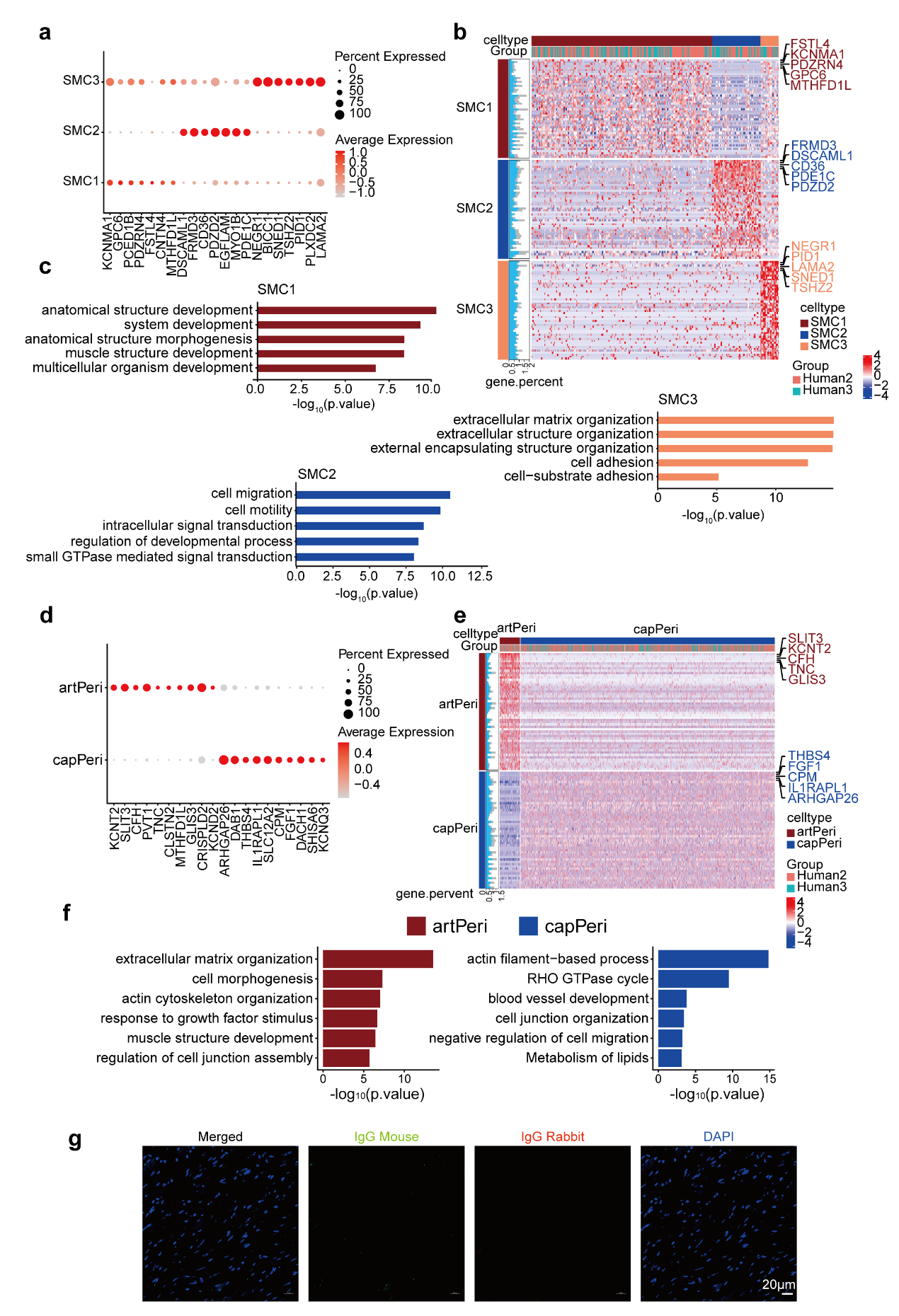


**Figure. S6. Smooth muscle cells (SMC) and pericytes subpopulations and gene regulation analysis.** (**a**) Marker genes of SMC sub-clusters. (**b**) Heatmap of differential expressed genes between SMC sub-clusters. (**c**) GO enrichment analysis of each SMC sub-clusters. (**d**) Marker genes of artery pericytes (artPeri) and capillary pericytes (capPeri). (**e**) Heatmap of differential expressed genes between artPeri and capPeri. (**f**) GO enrichment analysis of artPeri and capPeri. (**g**) IgG antibodies of the same species as negative control were used for staining of ABCC9 (mouse) and GLIS3 (rabbit).

**Figure. S7.**


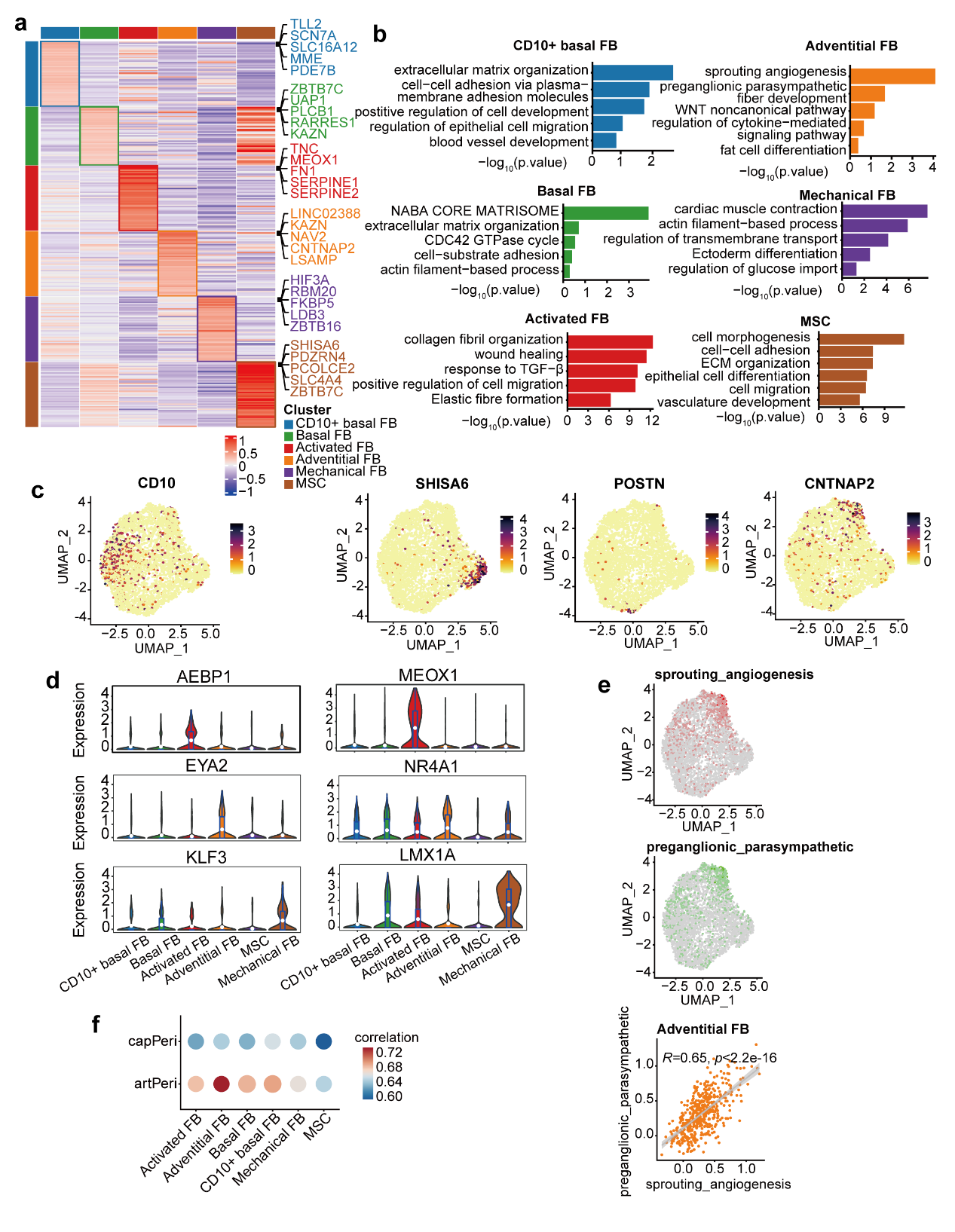


**Figure. S7. Fibroblast subpopulations and gene regulation analysis.**

(**a**) Heatmap showing the highly expressed gene signatures of each fibroblast subtype. (**b**) Enriched pathways of fibroblast subtypes. (**c**) Feature plot showing representative specific marker genes of fibroblast subtypes. (**d**) Expression of representative transcription factors (TFs) in fibroblast subtypes. (**e**) The expression levels of genes involved in sprouting angiogenesis and parasympathetic pathways and Spearman correlation of the cellular expression of genes involved in these two pathways. This analysis was used to demonstrate whether the cells associated with these two distinct pathways come from the same population or two subpopulations. (**f**) Spearman correlation of pericyte subtypes and fibroblast subtypes. Adventitial FBs showed the strongest correlation with arteriole pericytes (artPeris), suggesting synergistic effects in regulating vascular homeostasis.

**Figure. S8.**


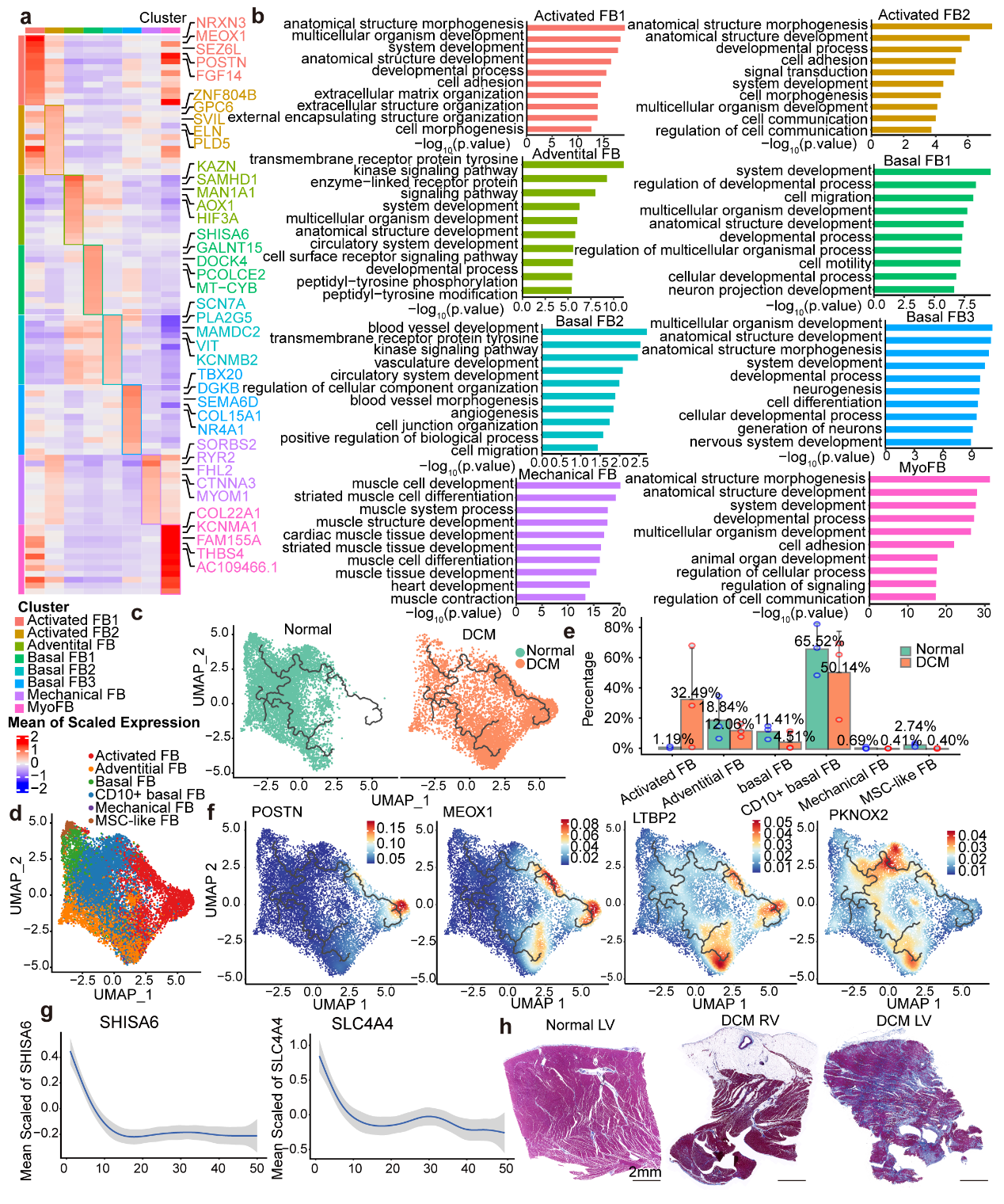


**Figure. S8. Subpopulations of fibroblasts in human donor and DCM hearts.** (**a**) Heatmap showing the highly expressed gene signatures of each fibroblast subtype according to the cell classification corresponding to Figure 6B. (**b**) Enriched pathways of fibroblast subtypes. (**c**) UMAP plot showing the difference of cell distribution between healthy and DCM hearts. (**d**) UMAP plot of fibroblasts featured by transfer label referring to the fibroblast classification of healthy hearts in Figure 5a. (**e**) The cell proportions of each transfer labelled cell subtype in healthy and DCM hearts, respectively. (**f**) Feature plot showing the expression levels of *POSTN*, *MEOX1*, *LTBP2* and *PKNOX2* along the trajectories. (**g**) The gene expression of SHISA6 and SLC4A4 along the trajectory branch 1 of fibroblasts from basal state to fibroblast activation then to fibrosis. (**h**) Masson staining of healthy left ventricle, DCM RV and DCM LV. Data is presented as the mean±SD. DCM, dilated cardiomyopathy; RV, right ventricle; LV, left ventricle.

**Figure. S9.**


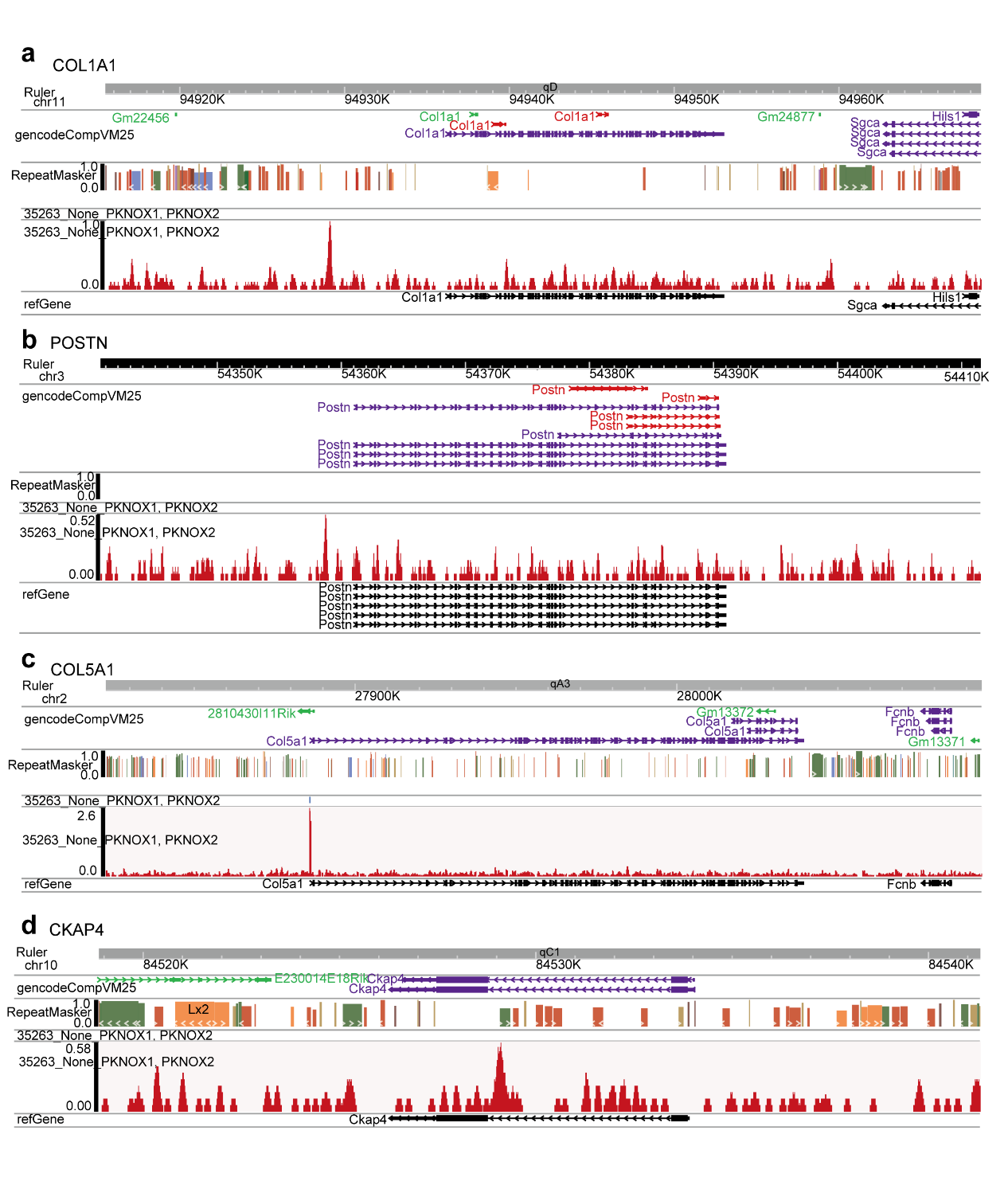


**Figure. S9. The transcription binding analysis of *PKNOX2* by published Chromatin Immunoprecipitation (ChIP) assay.** The *PKNOX2* directly binds to the promoter or intron regions of *COL1A1* (a), *POSTN* (b), *COL5A1* (c), and *CKAP4* (d), suggesting transcriptional regulation of these targeted genes by *PKNOX2*.

**Figure. S10.**


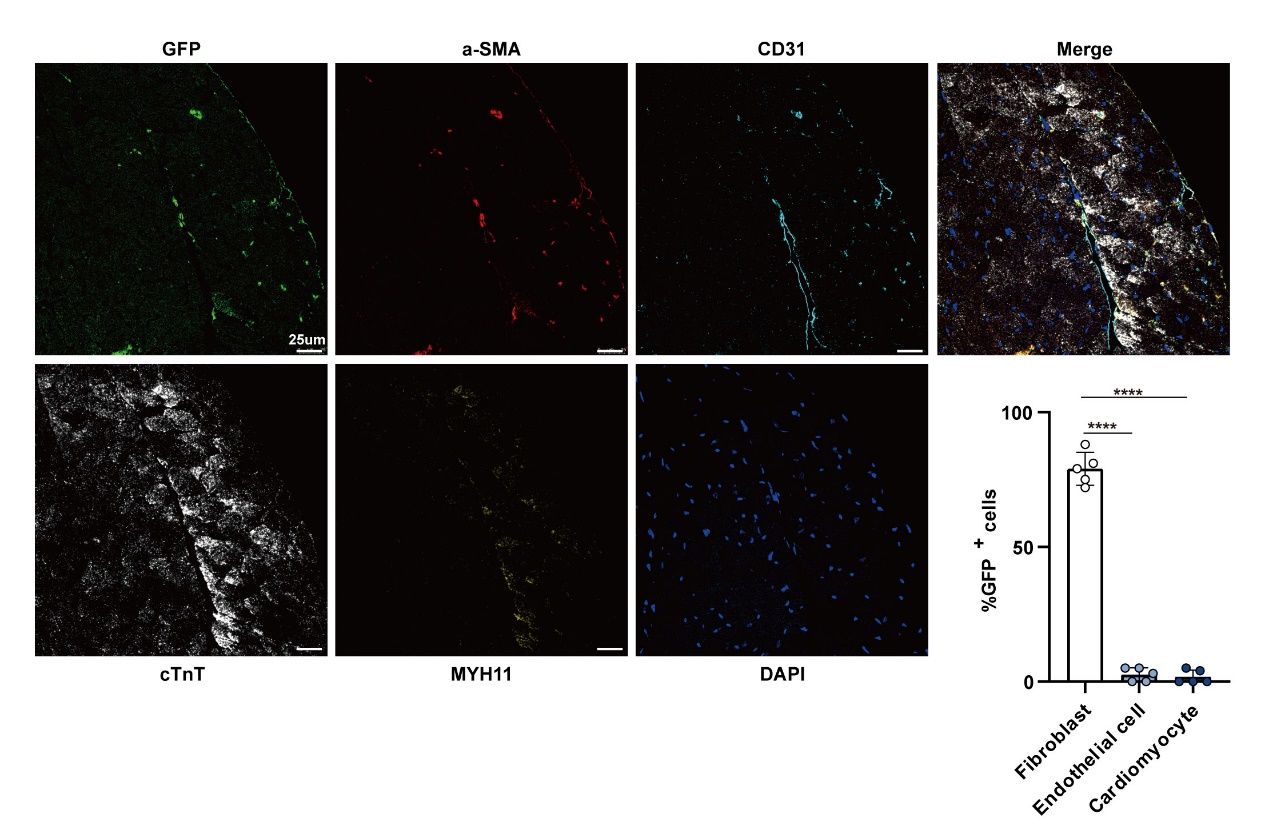


**Figure. S10.** **Overexpression efficiency and infection specificity of AAV9-Postn-Pknox2-GFP in adult mouse heart.**

Immunostaining shows the overexpression efficiency and infection specificity of AAV9-Postn-Pknox2-GFP in adult mouse heart. Number of GFP positive cells in different cell types. Cell marker: α-SMA, fibroblasts; CD31, endothelial cells; cTnT, cardiomyocytes; MYH11, smooth muscle cells. Data are represented as mean±SD. ****P < 0.0001.

**Table S1. (separate file)**

Human sample information

**Table S2. (separate file)**

Human heart celltype markers

**Table S3. (separate file)**

Normal EC subtype markers

**Table S4. (separate file)**

Human heart scRNA vs snRNA DE markers

**Table S5. (separate file)**

Human heart EC markers scRNA vs snRNA

**Table S6. (separate file)**

Human heart FB markers scRNA vs snRNA

**Table S7. (separate file)**

Normal Pericyte subtype markers

**Table S8. (separate file)**

Normal SMC subtype markers

**Table S9. (separate file)**

Normal FB subtype markers

**Table S10. (separate file)**

Normal FB trajectory DE markers

**Table S11. (separate file)**

DCM FB subtype markers

**Table S12. (separate file)**

DEG siPknox2 vs siCtrl

**Table S13. (separate file)**

DEG AAV9 Pknox2 vs AAV9 Control

**Table S14. (separate file)**

Marker genes of cardiac cell types

**Table S15. (separate file)**

List of siRNA sequences

**Table S16. (separate file)**

List of qRT-PCR primer sequences

**References**

1 Rao, M. *et al.* Resolving the intertwining of inflammation and fibrosis in human heart failure at single-cell level. *Basic Res Cardiol* **116**, 55 (2021).

2 McGinnis, C. S., Murrow, L. M. & Gartner, Z. J. DoubletFinder: Doublet Detection in Single-Cell RNA Sequencing Data Using Artificial Nearest Neighbors. *Cell Syst* **8**, 329-337 e324 (2019).

3 Wolock, S. L., Lopez, R. & Klein, A. M. Scrublet: Computational Identification of Cell Doublets in Single-Cell Transcriptomic Data. *Cell Syst* **8**, 281-291 e289 (2019).

4 Xi, N. M. & Li, J. J. Protocol for executing and benchmarking eight computational doublet-detection methods in single-cell RNA sequencing data analysis. *STAR Protoc* **2**, 100699 (2021).

5 Stuart, T. *et al.* Comprehensive Integration of Single-Cell Data. *Cell* **177**, 1888-1902 e1821 (2019).

6 Korsunsky, I. *et al.* Fast, sensitive and accurate integration of single-cell data with Harmony. *Nat Methods* **16**, 1289-1296 (2019).

7 Qiu, X. *et al.* Reversed graph embedding resolves complex single-cell trajectories. *Nat Methods* **14**, 979-982 (2017).

8 Cao, J. *et al.* The single-cell transcriptional landscape of mammalian organogenesis. *Nature* **566**, 496-502 (2019).

9 Wolf, F. A. *et al.* PAGA: graph abstraction reconciles clustering with trajectory inference through a topology preserving map of single cells. *Genome Biol* **20**, 59 (2019).

10 Tucker, N. R. *et al.* Transcriptional and Cellular Diversity of the Human Heart. *Circulation* **142**, 466-482 (2020).

11 Litvinukova, M. *et al.* Cells of the adult human heart. *Nature* **588**, 466-472 (2020).

12 Cui, M. *et al.* Dynamic Transcriptional Responses to Injury of Regenerative and Non-regenerative Cardiomyocytes Revealed by Single-Nucleus RNA Sequencing. *Dev Cell* **53**, 102-116 e108 (2020).

13 Reichart, D. *et al.* Pathogenic variants damage cell composition and single cell transcription in cardiomyopathies. *Science* **377**, eabo1984 (2022).

14 Li, H. *et al.* Optimized Langendorff perfusion system for cardiomyocyte isolation in adult mouse heart. *J Cell Mol Med* **24**, 14619-14625 (2020).

15 Alexanian, M. *et al.* A transcriptional switch governs fibroblast activation in heart disease. *Nature* **595**, 438-443 (2021).

16 Revelo, X. S. *et al.* Cardiac Resident Macrophages Prevent Fibrosis and Stimulate Angiogenesis. *Circ Res* **129**, 1086-1101 (2021).

17 Li, Y. *et al.* gp130 Controls Cardiomyocyte Proliferation and Heart Regeneration. *Circulation* **142**, 967-982 (2020).
